# Analysis of Isotopic Labeling in Peptide Fragments by Tandem Mass Spectrometry

**DOI:** 10.1371/journal.pone.0091537

**Published:** 2014-03-13

**Authors:** Doug K. Allen, Bradley S. Evans, Igor G. L. Libourel

**Affiliations:** 1 United States Department of Agriculture, Agricultural Research Service, Plant Genetic Research Unit, St. Louis, Missouri, United States of America; 2 Donald Danforth Plant Science Center, St. Louis, Missouri, United States of America; 3 Department of Plant Biology, University of Minnesota, St. Paul, Minnesota, United States of America; University of Westminster, United Kingdom

## Abstract

Phenotype in multicellular organisms is the consequence of dynamic metabolic events that occur in a spatially dependent fashion. This spatial and temporal complexity presents challenges for investigating metabolism; creating a need for improved methods that effectively probe biochemical events such as amino acid biosynthesis. Isotopic labeling can provide a temporal-spatial recording of metabolic events through, for example, the description of enriched amino acids in the protein pool. Proteins are therefore an important readout of metabolism and can be assessed with modern mass spectrometers. We compared the measurement of isotopic labeling in MS^2^ spectra obtained from tandem mass spectrometry under either higher energy collision dissociation (HCD) or collision induced dissociation (CID) at varied energy levels. Developing soybean embryos cultured with or without ^13^C-labeled substrates, and *Escherichia coli* MG1655 enriched by feeding 7% uniformly labeled glucose served as a source of biological material for protein evaluation. CID with low energies resulted in a disproportionate amount of heavier isotopologues remaining in the precursor isotopic distribution. HCD resulted in fewer quantifiable products; however deviation from predicted distributions were small relative to the CID-based comparisons. Fragment ions have the potential to provide information on the labeling of amino acids in peptides, but our results indicate that without further development the use of this readout in quantitative methods such as metabolic flux analysis is limited.

## Introduction

The harvested seeds of cultivated crops are an important source of protein, oil and carbohydrate used for food, feed and fuels. Investigations of plant cellular function and metabolic flux through biochemical networks are timely topics (e.g. [1]–[12]) due to the ever increasing global demand for plant-based commodities. Plant metabolism operates systemically [13], [14], in a time dependent fashion and across cellular [15]–[19] and subcellular levels of organization [20]–[24]. Accounting for these aspects is a prerequisite for biotechnology, including proper selection of promoters and targeting sequences in gene constructs. Yet, our understanding is incomplete due to purification methods that limit the subcellular and cellular resolution of metabolite pools.

Some of the complexity resulting from compartmentalization can be reduced through methods focused on subcellular organelles. For instance, traditional organelle fractionation protocols [25] have been updated in technique [26], [27] and instrumentation [28], [29] to minimize cross-contamination of metabolites and to improve sensitivity. For more abundant compounds, *in vivo* nuclear magnetic resonance (NMR) [30] can detect subcellular pH differences through changes in the chemical shifts associated with metabolites in different compartments [31]–[36]. Mass spectrometric measurements in combination with chromatography can isolate and quantify metabolites with high sensitivity and throughput and have recently become amenable to metabolism at the single cell [19], [37] and even organelle [38] levels.

In conjunction with labeled amino acids, mass spectrometry can further quantify metabolic events. In the field of proteomics, mass spectrometry is used to quantify amounts of protein to describe cellular metabolism (e.g. [39], [40]); to probe subcellular regions [41]; and to understand tissue development [42] in plants. Hybrid mass spectrometers that combine linear and orbital ion trap configurations are operated in series and provide parts-per-million mass accuracy [43] with attomolar sensitivity [44]. In combination with experimental methods such as SILAC (stable isotope labeling with amino acids) [45], [46], iTRAQ (isobaric tags for relative and absolute quantitation) [47], iCAT (isotope-coded affinity tag) [48], [49], or derivatives of these approaches [50], protein samples are isotopically tagged for mass separation and quantitatively compared.

Isotopic labeling studies of central carbon metabolism exploit the bond-breaking and -forming reactions of enzymatic steps that redistribute ^13^C-substrates into products, such as amino acids that are used for protein synthesis. Therefore, the ^13^C-labeling of living tissues results in amino acid isotopologues that can be quantified after protein hydrolysis. Most proteins are translated in the cytosol, with their amino acids coming from cytosolic pools. However, eukaryotes also contain proteins synthesized in mitochondria and chloroplasts. Compartment-specific translation provides an opportunity to examine amino acid labeling in different locations. GC/MS has been previously used with ^13^C-labeling to study subcellular metabolism through metabolites such as fatty acids [51], [52] or to monitor amino acid labeling with prior protein separation [53]. Cell wall and starch precursors can also be resolved through the use of labeling investigations [22], [51], [54]. Recent investigations that utilize isotopic labeling to investigate protein turnover [55], [56] or perturbed levels [57]–[59] prompt the investigation into the suitability of high resolution mass spectrometry for metabolic studies including direct [60] or indirect [61] amino acid analyses.

**Figure 1:**
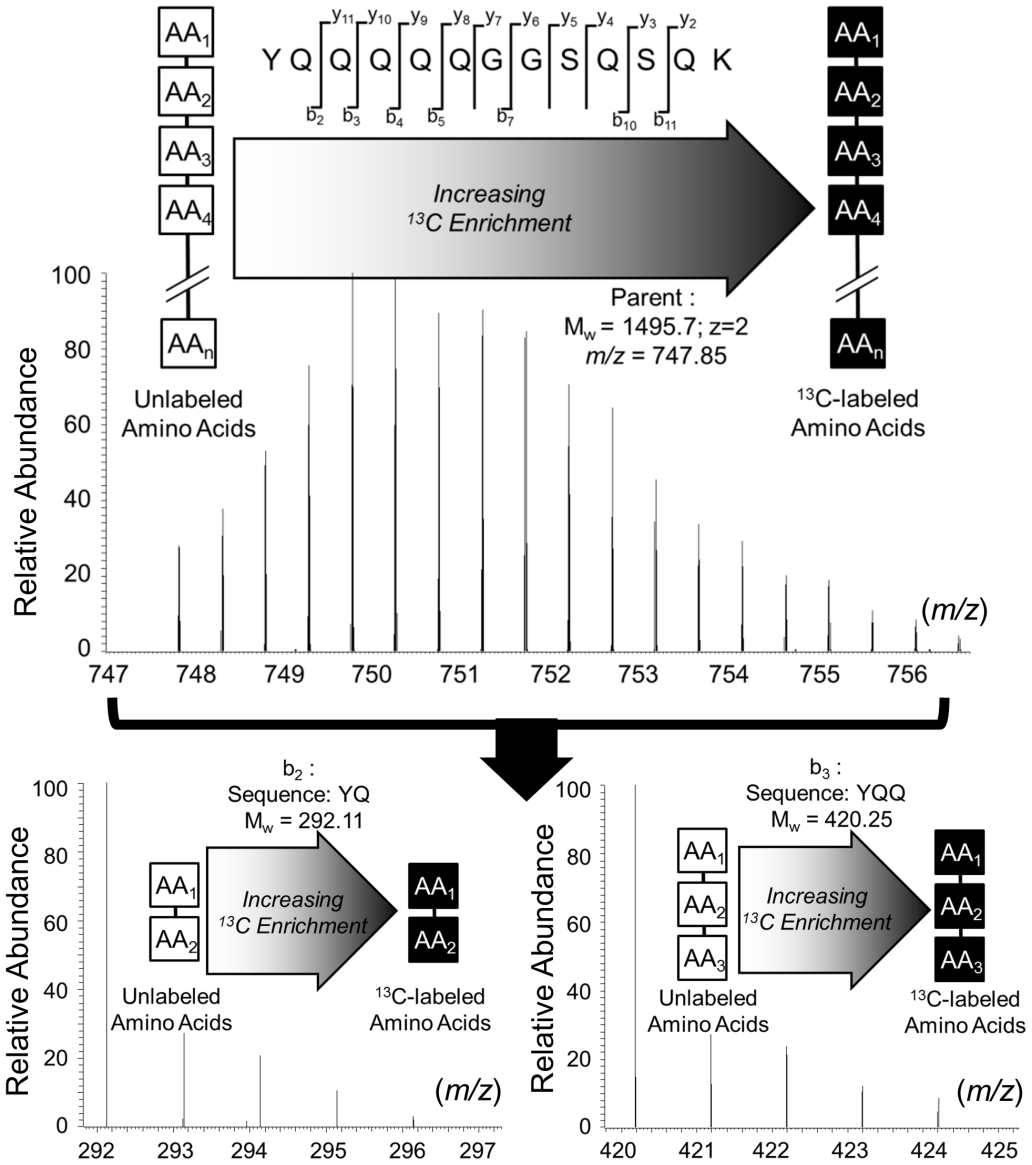
Analysis of peptides with tandem mass spectrometry. Labeled proteins result from either the heavy atoms present naturally or through culture with ^13^C labeled glucose. Proteolysis resulted in peptides that were fragmented by tandem mass spectrometry. Isotopic measurements resulted in quantitative labeling descriptions of sequences of amino acids.

Although quantification of peptides with isotopic shifts due to incorporated labeled amino acids is well-established, isotopic envelopes of peptides that result from multiple, partially labeled amino acids, have only recently been characterized [62], [63]. Furthermore, the accurate quantification of product ions resulting from fragmenting large isotopic envelopes has not yet been reported. Such methods are important because they can assess amino acid labeling within peptides that are tied to subcellular or cellular locations based on the origin of the protein. Additionally, because protein synthesis is a dynamic process, protein label also reports on the synthesis and turnover dynamics of individual proteins. Thus, the product ions from tandem MS describe labeling of a subset of amino acids (Figure 1) and product isotopologues have the potential to provide detailed information on metabolism.

We investigated the quantification of isotopes in *fragment ions* using high resolution orbital trap tandem mass spectrometry. Seven percent uniformly labeled *E. coli,* and unlabeled soybean cotyledons or those labeled with 100% [U-^13^C_6_] glucose in combination with unlabeled sucrose and amino acids served as a source of protein. Peptides from PAGE-isolated protein subunits digested with trypsin were fragmented and compared to predicted isotopologue distributions. CID and HCD methods of fragmentation were both investigated with multiple NCEs (normalized collision energies). Our results describe the energy required for fragmentation of parent peptides that are labeled. The CID process generated product ions with higher intensity than HCD but preferentially fragmented lower *m/z* parent isotopologues. In general, CID resulted in fragment ions with less than expected isotopic enrichments (i.e. the isotopic distribution of fragment ions exhibited a mass shift towards a lower *m*/*z* relative to the labeling pattern expected from the precursor ion). With increasing energies, HCD resulted in a decrease of MS^1^ ions and product ion abundances often went through a maximum value (i.e. considered as the sum of all product ions within a MS^2^ fragment isotopic envelope). HCD produced less significant deviations from the predicted isotopic distribution but resulted in lower abundances and fewer fragment ions could be quantified.

## Results

Developing embryos harvested from pods of soil-grown soybean plants were used for labeling investigations. Soybean biomass is composed of approximately 40% protein, including the two storage proteins glycinin and beta-conglycinin that comprise 50–65% of total protein. Small embryos (approximately 10 mg dry weight) were removed from pods and dissected from seed coats. Embryos were cultured for 14 days with sucrose and amino acids and [U-^13^C_6_]-glucose for the purpose of obtaining labeled material. Additional protein was obtained from direct harvest of green embryos without subsequent culturing. Seed biomass was lyophilized and pulverized in liquid nitrogen and prepared for SDS-PAGE as described in the methods. Prior to LC-MS/MS, individual bands were trypsin-digested, reduced, and alkylated as described in the methods. Unlabeled (samples or subunits) were first inspected by LC-MS/MS to determine retention times for peptides that were identified with the protein identification software Mascot v2.4. Both MS^1^ and MS^2^ spectra were recorded in the Orbitrap in profile mode. The retention times and monoisotopic *m/z* values for peptides were subsequently used to locate ^13^C-labeled isotopologues. *E. coli* was cultured in 7% uniformly labeled glucose (i.e. each carbon atom in glucose was 7% enriched) through multiple serial dilutions resulting in 9000-fold dilution of original culture volume and the generation of proteins that had a 7% atom percentage ^13^C incorporation level. These proteins were used to further assess CID-based quantification of product ions.

**Figure 2:**
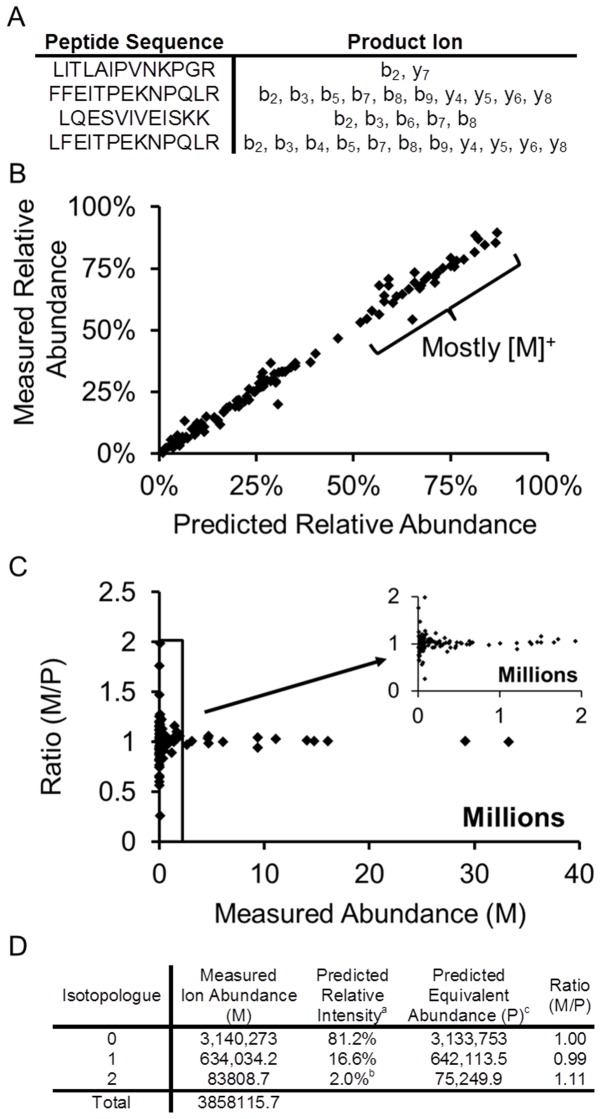
Analysis of fragment ions generated by HCD with NCE of 20%. A) List of peptides analyzed. B) Measured versus predicted relative abundances of natural isotopes in peptides. C) Ratio of measured isotopologue intensities. To account for the preponderance of monoisotopic ions present in unlabeled fragment ions, the intensity values were further considered. The abundances (M) within a measured *m/z* envelope were added and the summed value was multiplied by each of the predicted relative abundances resulting in abundances in intensity units for a theoretical distribution. The calculated abundance was subsequently compared to measured individual abundances through their ratio (M/P). If measured abundances matched the predicted values for each peptide, their ratio is unity. D) An example tabular form calculation of the ratio prepared in C. ^a^Calculated using natural abundance levels reported in [35] with given fragment elemental composition. ^b^ *m/z* values that could not be measured by the instrument because of low signal to noise were truncated, thus the sum is slightly less than 100%. ^c^Calculated as the Total Measured Ion Abundance multiplied by the Predicted Relative Intensity (e.g. 3,858,115.7×81.2% = 3,133,753).

Initially naturally abundant precursor isotopologues were surveyed after fragmentation with HCD with an NCE of 20%. The ten most intense ions from a full scan were subjected to MS/MS analysis. An isolation width of 3 *m/z* was sufficiently large to capture the entire isotopic distribution as confirmed by the MS^2^ spectra (i.e. inspection of the peptide indicated fragmentation was incomplete at 20% NCE and allowed monitoring in the MS^2^ spectra, additionally subsequent 0% NCE experiments confirmed isolation of the entire MS^1^ spectra by observation of the identical MS^2^ spectra). Twenty eight fragment ions from four precursors (Figure 2A) were individually quantified (Figures 2B and 2C). The isotopologues were compared to predicted values generated from the fragment elemental composition and reported natural abundance [64] (Figure 2B). In nature, heavy isotopes are present at low levels (e.g. abundance of ^13^C is approximately 1% of total carbon), thus the signal for isotopologues of low mass fragments (<1000 Da) at natural abundances is predominantly from monoisotopic ions. As the isotopic enrichment percentage (Figure 2B, x-axis) increases the monoisotopic peak is no longer dominant; the most-intense ion is represented by different isotopologues. Because the heavier isotopologues are present at low amounts in nature, they can be less sensitively measured and compared (Figure 2B).

The measured intensities of different peptide isotopologues are presented in Figure 2C. Measured intensities (M) for a given fragment were summed. The predicted abundances (P) were calculated from the sum of measured intensities and predicted relative abundances. These calculated intensities were compared to the measured values through their ratio (M/P) and plotted in Figure 2C with the calculation process summarized in Figure 2D. The isotopologues are indexed 0, 1, 2 to reflect the mass relative to the monoisotopic mass [i.e. (*m/z* of interest minus the monoisotopic *m/z*)*charge (z)]. This terminology was subsequently used for clarity throughout. Hence, instances in which the predicted and measured values were equivalent had a ratio of unity. Deviations from expectation were most significant at lower abundances (measured intensities of less than 100,000; Figure 2C). Figure 2 indicates that the relative abundances of the fragment ions examined were similar to the distribution predicted from elemental composition (many within 10% of the predicted value). We investigated specific fragment isotopologues and quantified them through orbital trap measurements for a more detailed assessment of the measurement accuracy and sensitivity.

**Figure 3:**
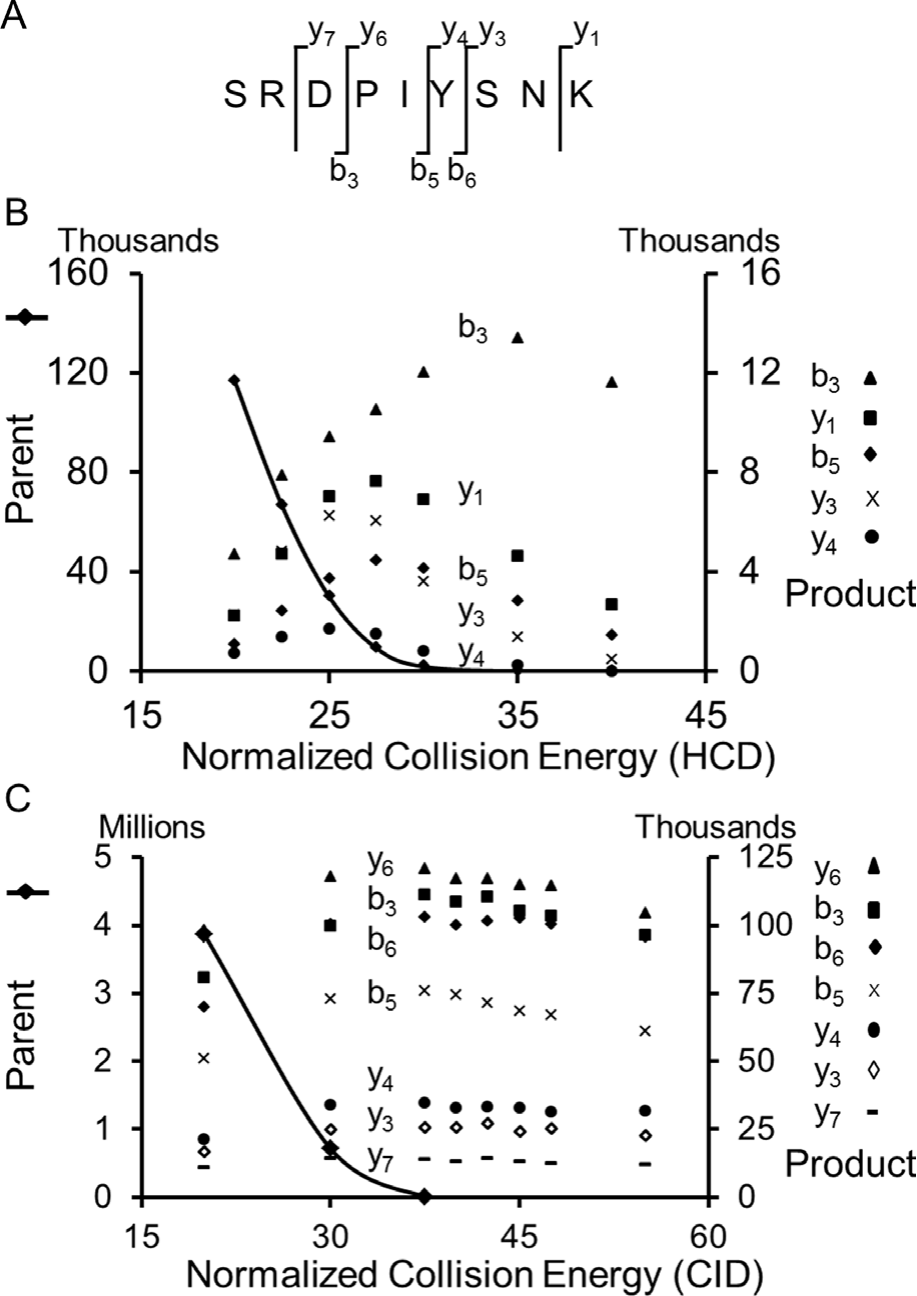
Measured abundance of precursor and fragment ions. Ions were measured as a function of A) higher energy collisional dissociation (HCD) energy and B) collision induced dissociation energy (CID). Each symbol represents a measured summed abundance for an isotopic distribution.

For the product ions to accurately represent the peptide labeling distribution, the fragmentation of the precursor ion isotopic distributions must break bonds in a randomized way. A bias in fragmentation could result in the over-representation of certain isotopologue groups, and the under-representation of others within product ion distributions. We inspected the degree to which the precursor ion isotopic distributions were fragmented by observing the remaining precursor ion in the MS^2^ spectra. HCD values were incremented from 2 to 95% of maximum NCE (Figure 3, File S1). CID fragmentation was similarly evaluated. Selected peptides were fragmented using an isolation window sufficient to capture the entire isotopic distribution, as was verified by comparison of MS^1^ to MS^2^ spectra. The abundances of parent (plotted as a line in Figure 3) and product ions (symbols) at various energies were determined and recorded as sums of the intensities of individual isotopologues within an isotopic distribution. Precursor abundance diminished with increasing NCE and was generally not observed in MS^2^ spectra at values greater than 35% (HCD) or 45% (CID) (Figure 3 and File S1). The summed intensities of fragment ions were higher for CID than HCD though intensities for HCD varied more extensively with NCE value. The more significant decrease in product ion abundance from higher HCD energies may reflect further collisional activation and fragmentation associated with beam-type CAD activation of HCD as opposed to resonant excitation of CID in an ion trap.

**Figure 4:**
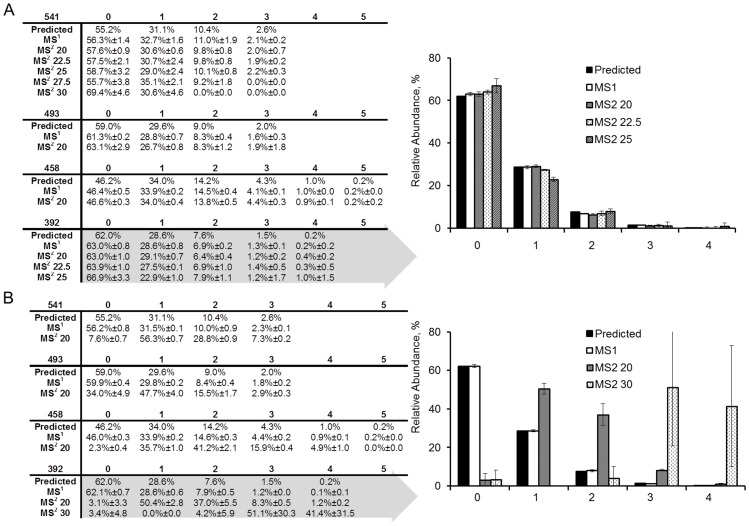
HCD and CID precursor ion isotopic distributions observed in MS^2^. A) HCD-derived values did not change significantly with fragmentation energy, whereas B) CID values were more variable and did not agree with prediction. The isolation windows were chosen to include the entire isotopologue set and were described by approximate m/z values (i.e. 392, 541 etc) subjected to fragmentation.

**Figure 5:**
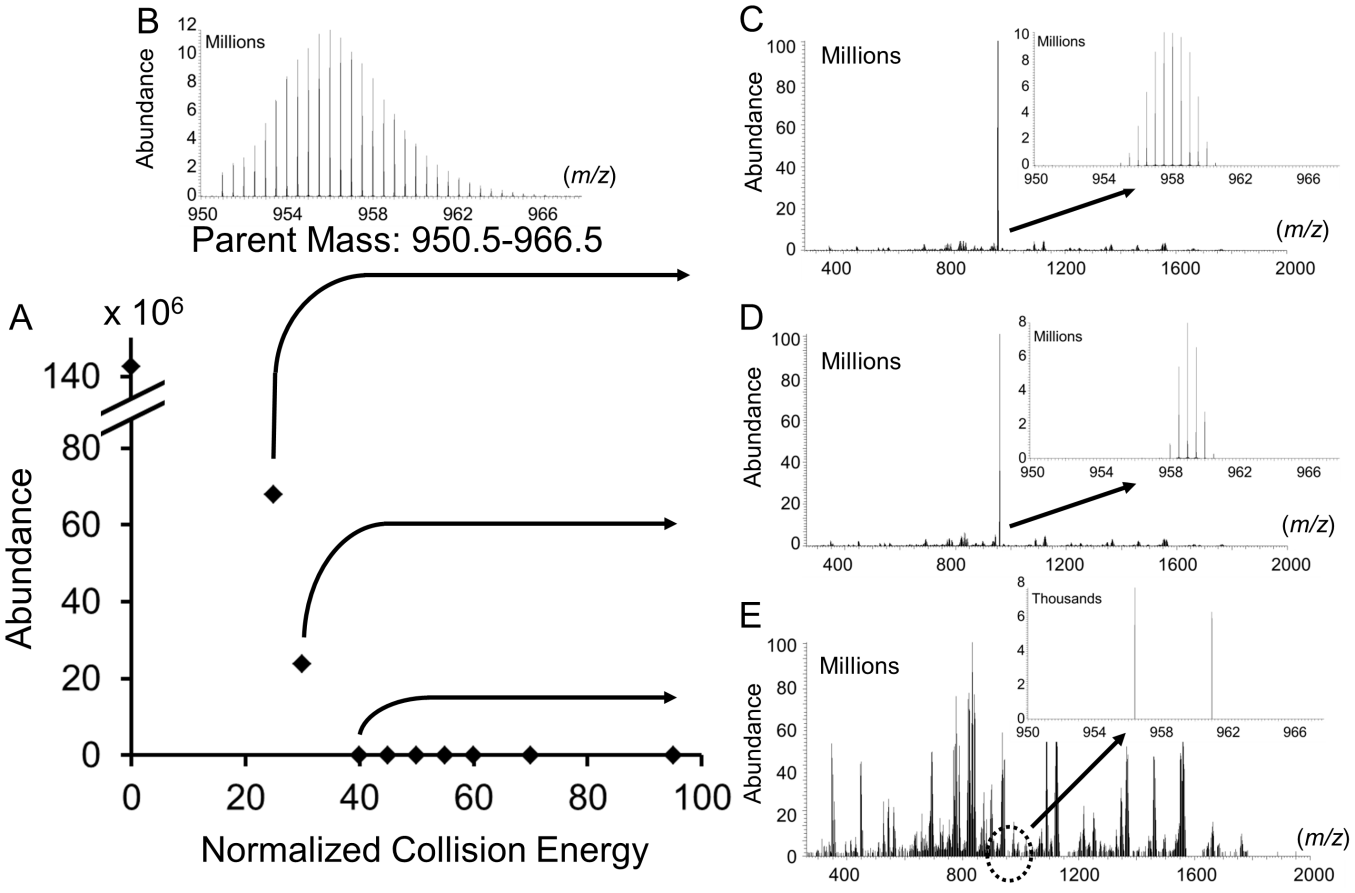
Comparison of the impact of different CID energies on fragmentation of ^13^C-labeled peptides. NCE’s approaching 40 are necessary to completely fragment the precursor ion. Reduced energies resulted in asymmetric fragmentation, reflected by the isotopic distributions skewed towards higher *m*/*z*. A) indicates the applied NCE to the precursor ion distribution B) that results in a remaining ion distribution that is skewed at multiple energies (C–E).

The isotopic distribution for each of four precursor ions (sequence, charge and observed *m/z* in File S2) were monitored by MS^1^ and again as the remaining precursor in MS^2^ after HCD (NCE of 20–27.5%, three measurements near the apex of the chromatographic peak served as technical replicates). Figure 4A indicates that the abundances measured in MS^2^ of the remaining precursor ion were similar to those predicted from elemental composition and reported levels of natural abundance [64]. At the greater fragmentation energies the relative abundances were less precise possibly as a consequence of reduced signal to noise. Figure 4B presents a similar analysis for CID spectra. The precursor isotopic distributions measured in MS^1^ were similar to the prediction as indicated by the table, whereas the remaining isotopic distribution in the MS^2^ spectra at NCE of 20 and 30% were distinct (measured through the MS^2^ spectra). The inset tables indicate the same was true for multiple peptides, each representing an independent measurement of the biased fragmentation. We also observed a similar consequence in much larger isotopic distributions such as those obtained with ^13^C-labeled peptides (Figure 5 and File S3). Figure 5A indicates the change in summed isotopologue abundance with CID energy for a ^13^C-labeled peptide. Figure 5B, 5C, 5D and 5E present the MS^1^ spectrum and remaining isotopic distributions monitored in MS^2^ with energies of 20–40% NCE respectively. File S3 provides a similar pattern for a different ^13^C-labeled peptide and this pattern was consistent across multiple samples from either labeled or unlabeled biomass. The spectra revealed that lower *m/z* values within an isotopic distribution were preferentially fragmented relative to the higher *m*/*z* isotopologues (e.g. absence of *m/z* 950–954 but presence of peaks around *m/z* 958 in Figure 5). Disappearance of the entire precursor isotopic distribution required a CID energy of 40% NCE (Figure 5E). The *m*/*z* and isolation windows subjected to CID were centered near the most intense ion and the isolation window was sufficient to capture the entire isotopic distribution.

**Figure 6:**
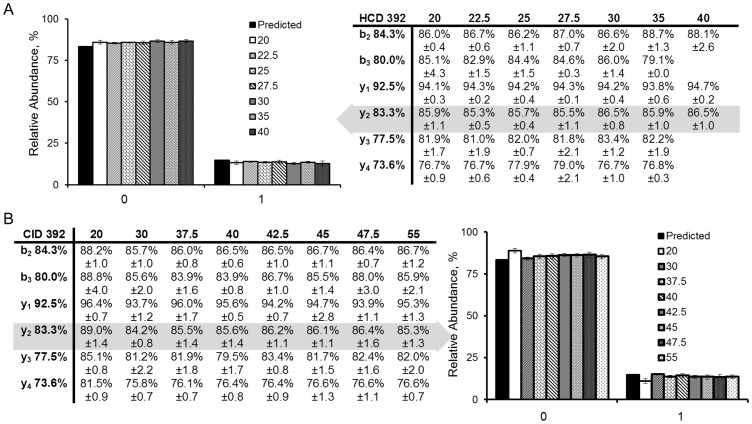
HCD and CID relative abundances of fragment ion isotopic distributions. A) HCD produced spectra that did not change extensively with increasing fragmentation energy, whereas B) incomplete CID fragmentation of the precursor resulted in skewed isotopologue abundances at low energies. Predicted values from natural abundance are indicated in bold for each fragment.

We hypothesized that the CID energy used to fragment peptides could result in bias in the MS^2^ spectra. The product isotopologue intensities for both CID and HCD were quantified in Figure 6. The isotopologues from b and y ions were integrated through multiple scan events (n = 3) for all four peptides at multiple HCD and CID values and monoisotopic mass (0) (Figure 6 and inset table) and singly labeled (1) (Figure 6) were compared to predicted values from natural abundance. The predicted value of the monoisotopic relative abundance is presented in bold and compared to measured values at different HCD energies. HCD produced isotopic distributions similar to calculated values for natural abundance (Figure 6A) but the monoisotopic peak was overestimated relative to the predicted value. The observation did not change as a function of the HCD energy and was consistent across peptides. Qualitatively, a similar trend was true for CID, but the agreement between measured and calculated isotopologue abundances from CID was weaker at lower energy. The lowest CID energy (NCE of 20%) yielded the most divergent isotopic distribution comparison with a significant overestimation in the monoisotopic peak intensity compared to natural abundance. At this CID energy, some of the precursor ion had not been fragmented (e.g. Figure 4B, Figure 5) and comprised a higher relative percentage of heavy isotopologues.

**Figure 7:**
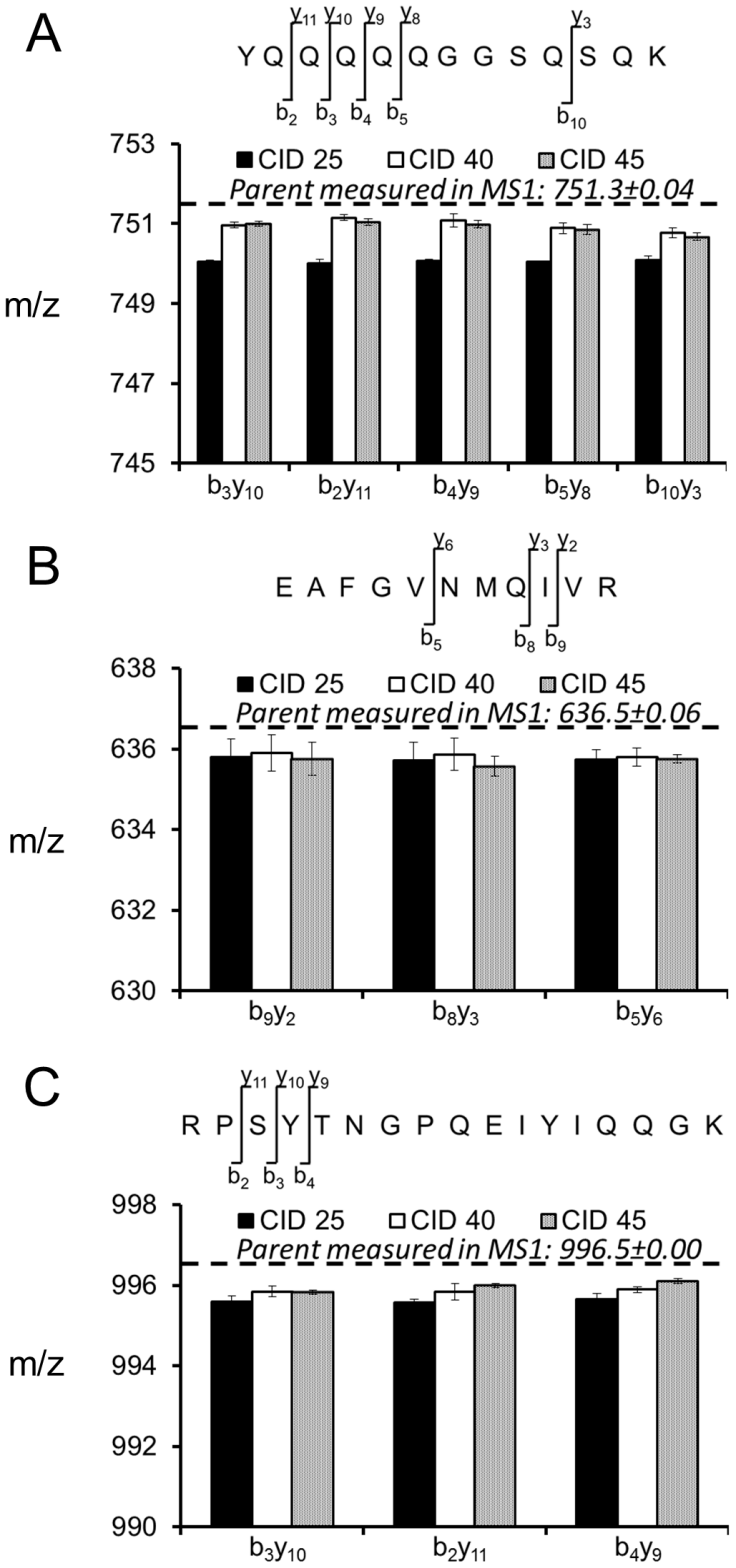
The average *m/z* value of the precursor isotopic distribution was measured and additionally calculated as the sum of two product isotopic distributions. A), B) and C) are different peptides evaluated at three CID values. The average *m/z* value calculated from the sum of product ions was an underestimate of the value calculated from the MS^1^ spectra.

Differences in predicted and measured fragment isotopic distributions were also observed in ^13^C-labeling experiments that produced wider isotopic distributions. The first involved isotopically labeled soybean embryos. Unlike peptides with isotopes present at well-described natural abundance levels, the exact isotopic composition for isotope-labeled soybeans could not be predicted a priori and the comparison of the average isotopic enrichment in the precursor was compared to two complementary fragments (e.g. b- and y-ions from cleavage at a single peptide bond). The average *m/z* defined as the sum of envelope abundances multiplied by their respective *m/z* values, divided by the sum of abundances (i.e. Σ[abundance×*m/z*]/Σ[abundance]) was calculated for precursor and fragment ion distributions. The average *m/z* value of the parent should be equivalent to the average *m/z* values for the sum of the two product ions that comprise the parent (e.g. parent with 11 amino acids should have an average *m/z* equivalent to the paired average *m/z* values for b_4_ and y_7_, or b_2_ and y_9_, or b_6_ and y_5_ etc.). For this comparison the mass spectrometer isolation window was intentionally set to a large value of 35 *m*/*z* and CID energies of 25, 40 and 45% NCE were chosen based on prior results. The average *m/z* for parents and sum of product ions were calculated in triplicate using the three scans nearest the apex of their respective chromatographic peak for each of three peptides and are presented in Figure 7. In every case the fragment ion isotopic distributions underestimated the labeling in the measured precursor ion as indicated by the lower average *m/z* value consistent with observations from natural abundance peptides. As the peptides were significantly labeled (e.g. Figure 5 contains abundant isotopologues that are distributed amongst many intermediately labeled masses), the underestimated average *m/z* values were not a consequence of lower signal to noise for high mass isotopologues in the MS^2^ spectra (Figure 7).

The bias in labeling was further inspected through 7% uniformly labeled *E coli* peptides. A list of peptides was generated from unlabeled protein of *E. coli* cultured in M9 media. For peptide identification, unlabeled protein was digested by trypsin, treated and analyzed by MS as before. Peptides eluting at distinct retention times were quantified with both orbitrap and linear ion trap across a range of CID energies that further confirmed the bias (File S4). The provision of 0% NCE produced intact peptides observed in MS^2^ containing approximately 7% atom labeling on average. Product ions at various NCE values were lighter (File S4). The results indicate that the average labeling measured in product ions is dependent on the fragmentation energy but that y and b fragments are not biased differently from one another.

**Figure 8:**
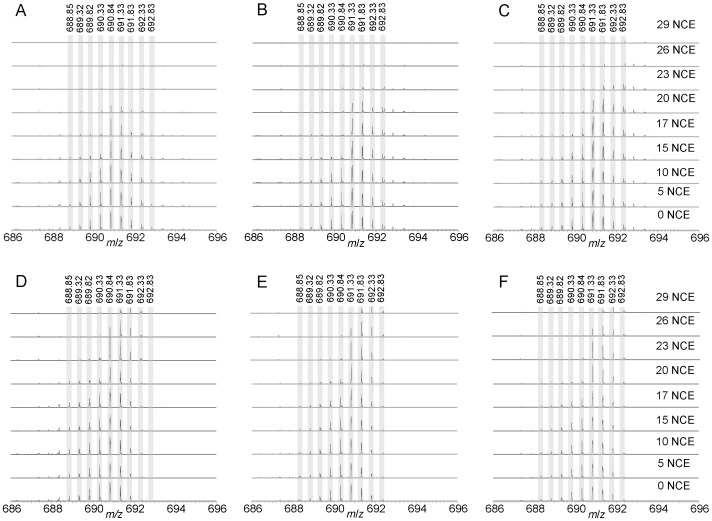
The bias toward preferential fragmentation of lower *m*/*z* precursor isotopologues in CID is independent of chromatographic or space-charge effects. Seven percent ^13^C labeled *E. coli* tryptic digest was directly infused into the mass spectrometer. The doubly charged ion of A46-K57 of EF-Tu1/EF-Tu-2 at *m*/*z* 690.84 was targeted for CID MS^2^ at NCE values of 0, 5, 10, 15, 17, 20, 23, 26, and 29 at AGC targets of A) 10,000, B) 50,000, C) 100,000, D) 500,000, E) 1,000,000 and F) 5,000,000. Through monitoring of the remaining precursor isotopic distribution in MS^2^ scans it is evident that there is a bias towards preferentially fragmenting lower *m*/*z* precursor isotopologues.

To investigate if the underestimation of fragment masses was a function of the ion density in the orbital trap, the automated gain control (AGC) target values were systematically varied between 10,000 and 5,000,000 at collisional energies from 0 to 29 NCE (Figure 8) while directly infusing a 7% ^13^C labeled *E. coli* sample. The doubly charged ion of A46-K57 of EF-Tu-1/EF-Tu-2 (File S5) at *m*/*z* 690.84 was selected for MS^2^. We found that there was a bias towards fragmentation of lower *m*/*z* precursor isotopologues regardless of the number of ions in the ion-trap during the CID process. The bias was not a consequence of ion choice or isolation window as fragmentation was centered very near the base peak and the isolation window sufficient to include the entire isotopologue distribution.

## Discussion

One of the current limitations to systems level explorations in eukaryotes is the lack of information about cellular heterogeneity, subcellular organelles and temporal dynamics of metabolism. Proteins that are made at specific times and from genomes of organelles in distinct locations, encode such information. In the past we have evaluated differences in labeling of amino acids as a result of subcellular location [53], but more general methods and enhanced sensitivity will add to the versatility of this approach. There are now reports on the application of mass spectrometry at the single cell [37], and subcellular [28], [29], [38] levels to probe metabolite levels, and a large number of proteomic studies that quantify protein turnover or relative protein levels with the aid of isotopic labeling methods. We investigated if the isotopic profiles of MS^2^ peptides that result from MS^1^ fragmentation in tandem mass spectrometry provided data consistent with isotopologue predictions and what fragmentation form and energy is appropriate for quantification.

### Available Information in MS^2^ Fragmentation

There are several distinctions between the indirect measurement of amino acid labeling through peptides and the direct measurement of amino acid enrichment. The preparation of amino acids for direct analysis by GC or LC-MS involves hydrolysis of peptides, which degrades some amino acids through oxidation (cysteine, tryptophan) or deamidation (glutamine, asparagine). Additionally, standard methods for GC-MS analysis, include prior tert-butyldimethylsilyl derivatization, which: 1) create complex fragmentation patterns (arginine) [65], [66]; 2) have amino acids with lower abundances (histidine); and 3) exhibit interference from contaminating compounds (e.g. compounds that co-elute with proline) [67]. Finally, though peptides are synthesized in specific locations utilizing organelle specific genomes, this information is lost when total cellular protein is collected and hydrolyzed in bulk.

GC-MS analysis yields multiple isotopic readouts because fragmentation occurs between carbons within the backbone. Similarly, fragmentation with a particular dissociation technique (i.e. CID, HCD, ETD) has consequences for the point of cleavage within the backbone, thus the use of multiple energy forms provides complementary information. Electron transfer dissociation methods are capable of producing c and z ions [68], whereas CID/CAD/HCD more commonly produces b- and y-type fragment ions [69], which were evaluated here. Additional information could be obtained from d, d’, w and w’ ions that occur with fragmentation in the side chain of some amino acids (also called satellite ions [70]), but are less frequent. Therefore labeled peptides have the potential to provide distinguishing information on metabolism through the indirect measurement of amino acids.

### Consequence of Fragmentation Energy on MS^1^ and MS^2^ Abundance

The CID energy had an unexpected effect on the observed fragmentation patterns. Low CID energies resulted in incomplete fragmentation of precursors. The remaining precursors’ isotopic distributions were skewed towards higher *m*/*z* (Figure 4B, 5, File S3). HCD also resulted in peptides that were incompletely fragmented, but with isotopic distributions comparable to predicted values (Figure 4A). At higher energies, both HCD and CID produced product ion isotopic distributions that were similar to predicted values, but the measured values consistently overestimated the low *m/z* intensities at the expense of others for reasons unknown (Figure 6). Inspection of the ratio of measured intensities for fragment ion isotopologues (File S6) gave a similar result, indicating the difference was not a reflection of low signal to noise though we (File S7) and others [71] have noticed more variability in lower abundance fragments. When we inspected the MS^1^ spectra for similar errors (File S2), the predicted and measured values were not statistically different. Additionally, changes in isolation width (File S8) did not bias quantification and small variation in approximating natural abundance also could not account for the observed results. Furthermore, the observed fragmentation bias was independent of the ion density in the trap, indicating that the observed bias was unrelated to possible space-charge effects [71]. Experiments with 0% NCE resulted in MS^2^ spectra that could not be distinguished from MS^1^ indicating that precursor isolation, excitation or subsequent ion transmission did not result in the observed bias. We also examined b and y-ions to see if the cleavage of the precursor resulted in consistently more labeled b or y-ions due to the isotope dependent difference in energy requirements for carbon bond breakage (zero-point energy effect, File S4). However, our results do not support such a chemical kinetic isotope effect.

The apparent preferential fragmentation of lower *m/z* isotopologues as evident by incomplete fragmentation of the higher *m*/*z* precursor isotopologues, suggests that fragmentation was *m/z* dependent. This finding was consistently observed through multiple experiments with or without isotopic labeling in both *E*. *coli* and soybean embryos. These observations suggest that a product ion isotopic distribution may not accurately represent the underlying isotopic composition of the intact peptide, and the use of fragments for quantitative methods such as metabolic flux analysis may require further corrections or considerations.

## Materials and Methods

### Materials

#### Chemicals

[U-^13^C_6_]-glucose, and unlabeled sucrose, glucose, glutamine, asparagine plant protease inhibitor cocktail, Ponceau-S, trypsin and all common buffer reagents were purchased from Sigma (Milwaukee, WI). SDS-Page reagents were purchased from Invitrogen, (Carlsbad, CA). MTBSTFA +1% TBDMCS were purchased from Thermo Scientific (Waltham, MA). Uniformly labeled 7% glucose was obtained as a custom order from Cambridge Isotope Laboratories.

### Methods

#### Embryo Culture

Soybeans (Glycine max cv. Jack) were grown in a green-house under summer-like conditions (14/10 light/dark cycle, daily watering and fertilization, temperature 75–80°F with other conditions described elsewhere [72]. Developing pods were harvested and immediately placed on ice and surface sterilized with 5% bleach. Aseptic dissection of embryos ranging in size from 25–40 mg (wet weight) was followed by culturing with U-^13^C glucose (20% of total hexose) and other substrates including 150 mM sucrose, 75 mM glucose, 45 mM glutamine, 16 mM alanine, 5 mm MES pH 5.7 with KOH. Vitamins and salts were added as described [73], [74] before filter sterilization. Embryos were cultured for two weeks with constant 30 micromoles/m^2^/s light at 27°C.

#### Protein Preparation

SDS-PAGE gel reagents were purchased from Invitrogen (Carlsbad, CA). Soybean protein was separated by 4–12% Nu-PAGE gel with MES running buffer at 200 V constant for 35 minutes. Molecular weight was determined with SeeBlue-2 molecular weight markers (Life Technologies, Grand Island NY). Soybean biomass (approximately 3–5 mg) was suspended in 1 ml of sample Laemmeli buffer containing 5% beta-mercaptoethanol and boiled for 10 minutes. Twenty microliter samples were run in multiple lanes. Samples were visualized with Simply Blue Stain. Bands were excised, cut into 3–4 pieces and placed into separate plastic tubes. Slices of the gel were destained with 50% acetonitrile followed by 50% acetonitrile/100 mM ammonium bicarbonate until clear. Slices were then dried with a short incubation with 100% acetonitrile and a short time in a speed vacuum.

Slices from the gel were reduced with 50 µl of 10 mM dithiothreitol in 100 mM ammonium bicarbonate and then incubated for 30 minutes at room temperature (RT). Following removal of the supernatant, 50 µl of fresh iodoacetamide (55 mM in 100 mM ammonium bicarbonate) was added and incubated for 30 minutes at RT. Next the band pieces were washed with 100 mM ammonium bicarbonate followed by 20 mM ammonium bicarbonate/acetonitrile (1∶1). Band pieces were incubated in 100% acetonitrile followed by vacuum drying. Trypsin was added to the dried gel pieces (approximately 1∶10–1∶20 w/w, 0.15 micrograms total in 100 microliters 50 mM ammonium bicarbonate) and incubated overnight at 37 °C. The reaction was quenched by addition of 50 µl 1% formic acid +2% acetonitrile. A 50% acetonitrile solution was used to extract additional peptides from the gel. The supernatant extracts were combined and concentrated by drying in a separate vial.

#### Liquid Chromatography and Mass Spectrometry

Samples were analyzed using a LTQ-Orbitrap Velos mass spectrometer (Thermo Fisher Scientific, San Jose, CA, USA) coupled with a nanoLC Ultra (Eksigent, Dublin, CA USA). Samples (5 µl) were loaded onto a trap column (C18 PepMap100, 300 µm ×1 mm, 5 µm, 100 Å, Dionex, Sunnyvale, USA) at a flow rate of 4 µL/min for 5 min. Separation of peptides was conducted using a reversed phase C18 column (Acclaim PepMap C18, 15 cm×75 um×3 um, 100 A, Dionex) at a flow rate of 0.26 µL/min.

The separation employed a 38 minute linear gradient ranging from 2–65% B (mobile phase A: 0.1% formic acid; mobile phase B: 0.1% formic acid in acetonitrile). The mass spectrometer was operated in positive ion mode utilizing a full scan in the FT cell from *m/z* 50–2000, with resolution set at 60,000 at 400 *m/z*, in the profile acquisition mode and with automatic gain control (AGC) set to a target of 1,000,000 ions. HCD and CID energies were varied as described in the text. CID and HCD scan events were timed and targeted based on previously acquired data-dependent acquisition.

For direct infusion experiments, sample was infused using a Triversa chip-based nano ESI robot (Advion, Ithaca NY). Data were acquired over two minutes for each experiment while maintaining a spray current of 250 nA +/−20 nA. Scan events cycled between a full scan (*m*/*z* 400–2000) and CID MS^2^ scans targeting doubly charged A46-K57 of EF-Tu-1/EF-Tu-2 (NP_417798 or NP_418407.1, File S4) at *m*/*z* 690.84 with a 10 *m*/*z* isolation window at 0, 5, 10, 15, 17, 20, 23, 26 and 29 NCE (*m*/*z* 185–2000). One experiment consisted of the described scans summed over 2 minutes for each AGC target of 10,000, 50,000, 100,000, 500,000, 1,000,000 and 5,000,000. The maximum ion injection time was set to 8000 ms to allow for the instrument to achieve the desired fill target in all experiments. All scans were measured at a resolution setting of 60,000 in positive profile mode.

#### Data Analysis

Data were processed using Mascot Distiller v2.4 and searched using Mascot Daemon (Matrix Science, London, U.K.). Peptides were grouped and ranked by homology using Scaffold v3.1 (Proteome Software, Portland, OR USA). Identification of peptides was based upon NCBI library using a trypsin cleavage pattern, 0.80 Da and 15 ppm fragment and parent tolerances, respectively. Fixed modifications of +57 (carbamidomethyl) and variable modifications (−18 [pyro-glutamate], +1 [glutamine/asparagine deamidation], +16 [methionine oxidation] were all included in the Scaffold method. Integrated values of isotope incorporation were extracted from raw files manually and subsequently quantified as relative isotopologues.

## Supporting Information

File S1 **Inspection of Fragmentation Energy.**
(DOCX)

File S2 **Peptide Sequence and Precursor Data.**
(DOCX)

File S3 **Incomplete CID Fragmentation.**
(DOCX)

File S4 **FTMS and ITMS Comparison.**
(DOCX)

File S5 **EF-Tu Peptide used to Probe Automated Gain Control.**
(DOCX)

File S6 **Comparison of the Ratio of Measured Fragment Isotopologues.**
(DOCX)

File S7 **Fragment Abundances Contribute to Variability.**
(DOCX)

File S8 **Impact of Isolation Window on Isotopic Measurements.**
(DOCX)
