# Supplementary material for: Analysis of Isotopic Labeling in Peptide Fragments by Tandem Mass Spectrometry
Source: PLoS One. 2014 Mar 13;9(3):e91537. doi: 10.1371/journal.pone.0091537 (PMC3953442; doi:10.1371/journal.pone.0091537)

**Analysis of isotopic labeling in peptide fragments by tandem mass spectrometry**

**Doug K. Allen*, Bradley S. Evans and Igor G. L. Libourel**

**File S1: Inspection of Fragmentation Energy**

Additional spectra were investigated to determine the energy necessary for complete fragmentation of peptides with HCD or CID. The unfragmented precursor ion that remains is indicated by the line. Fragment ion isotopic distributions are presented as unconnected points. Each point is the average of at least 2 technical replicates. Intensities are plotted.


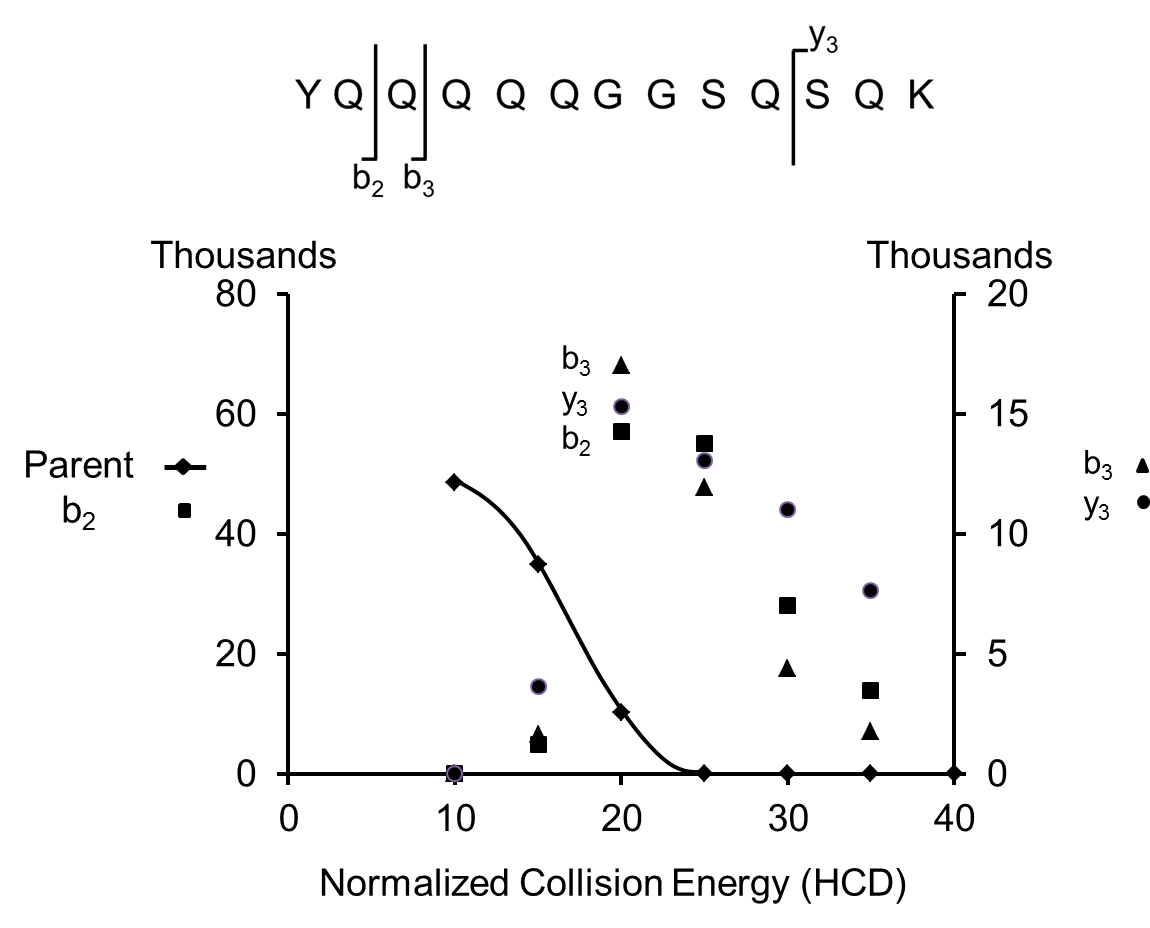


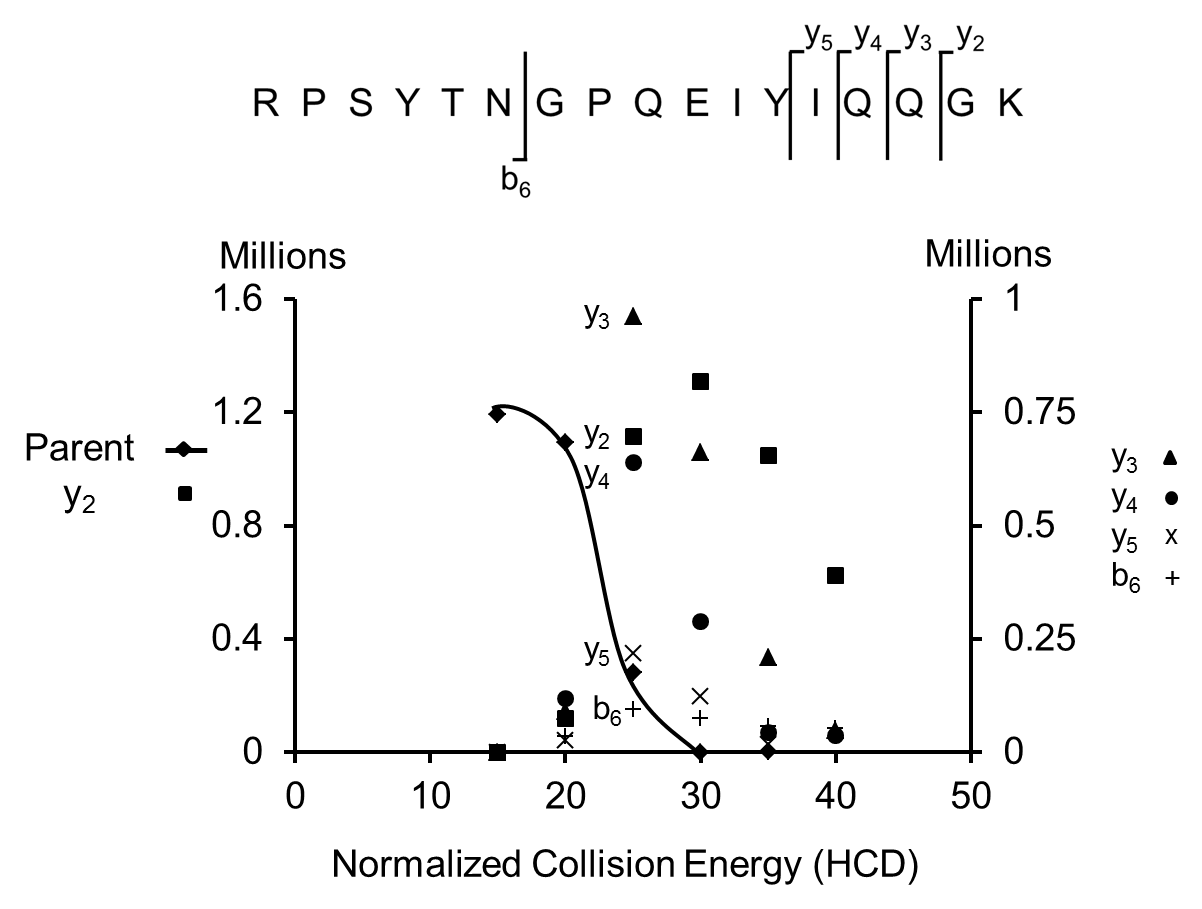


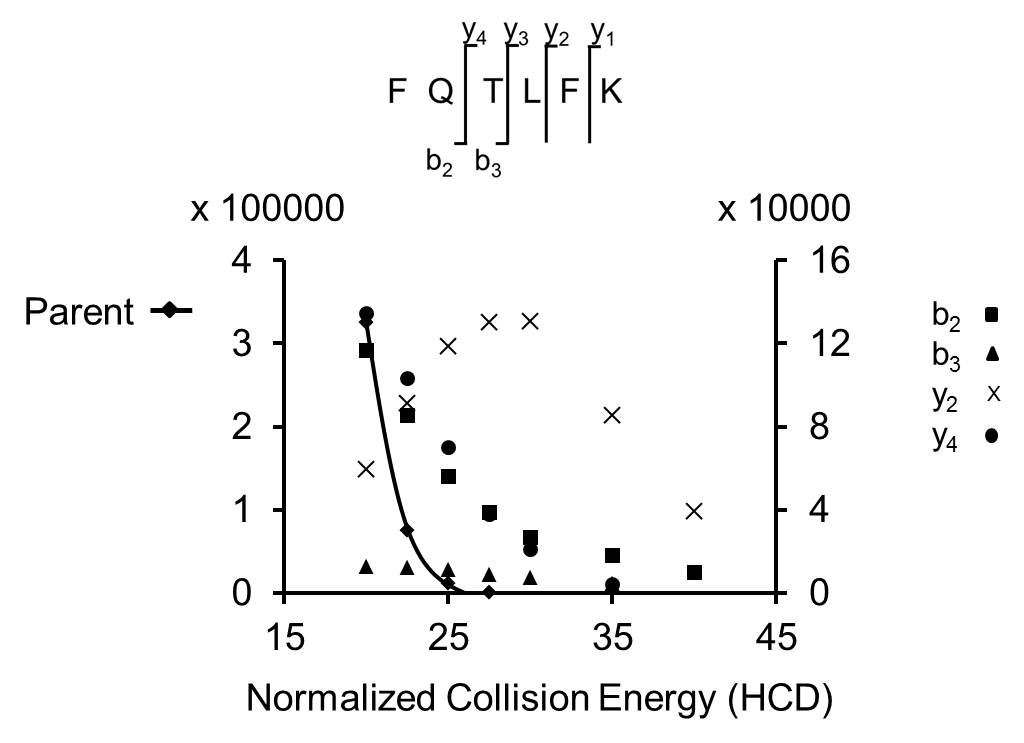


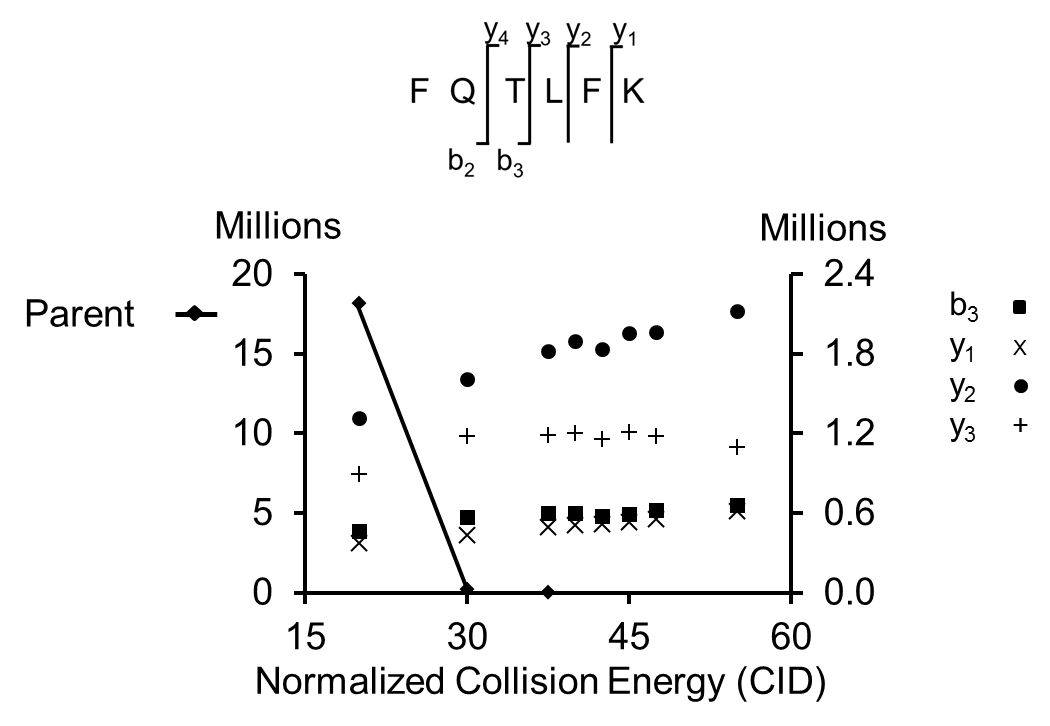


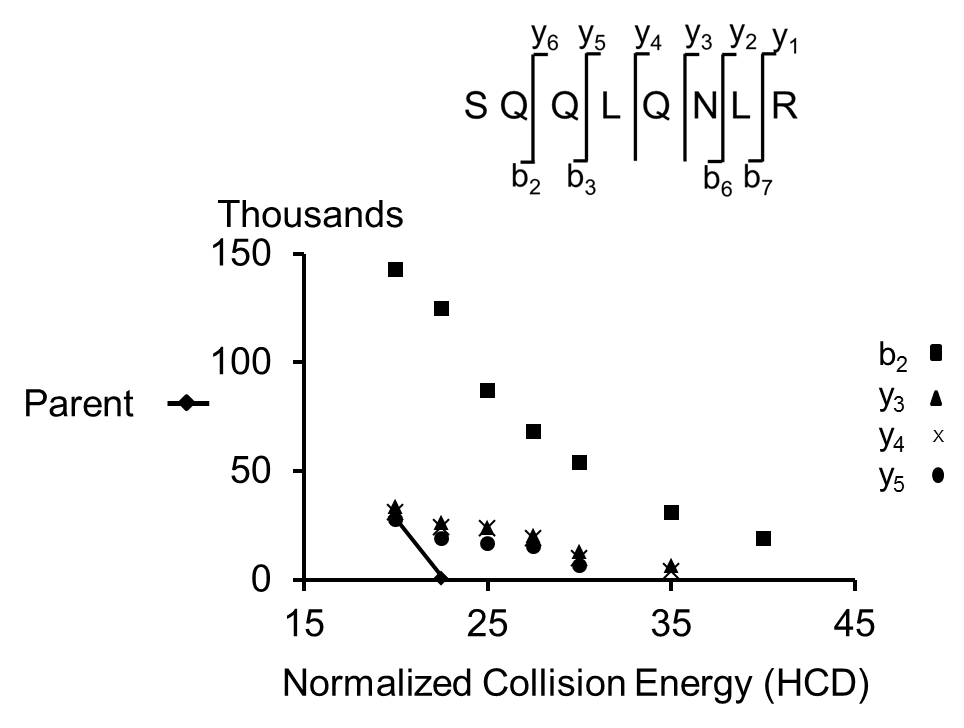


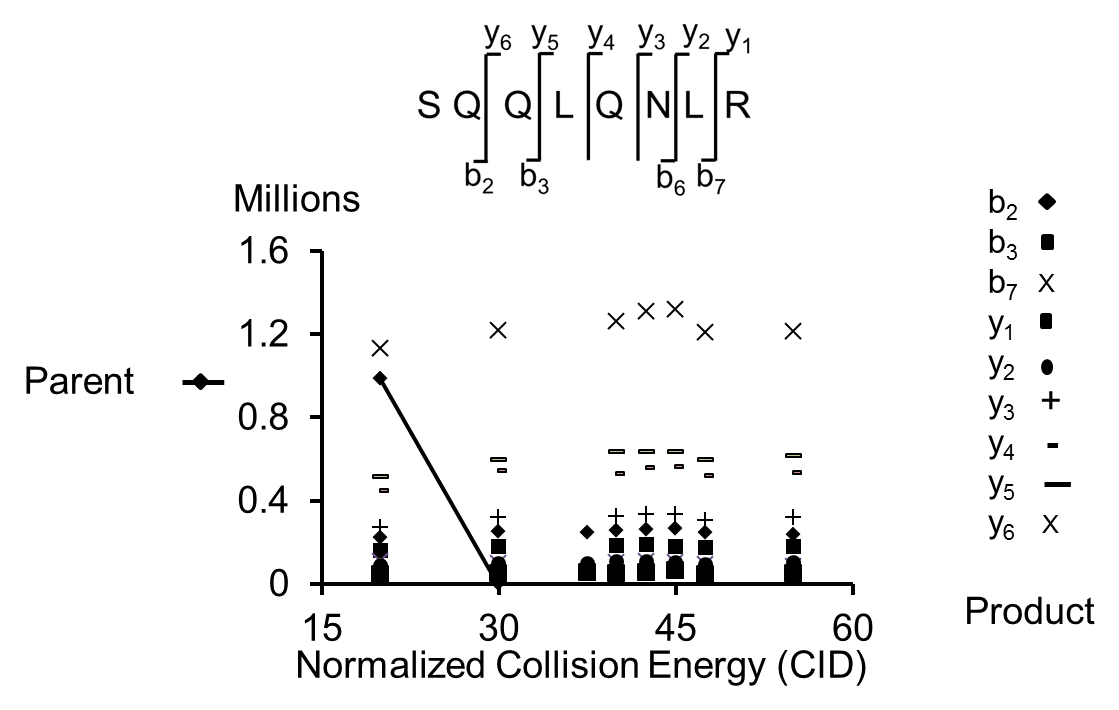


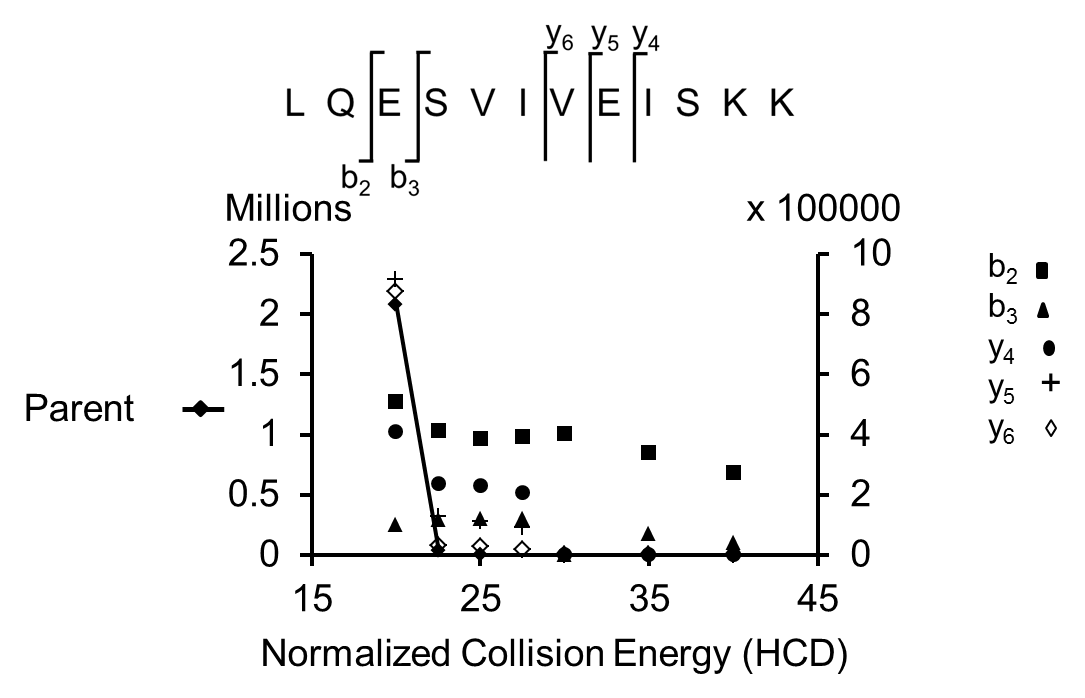


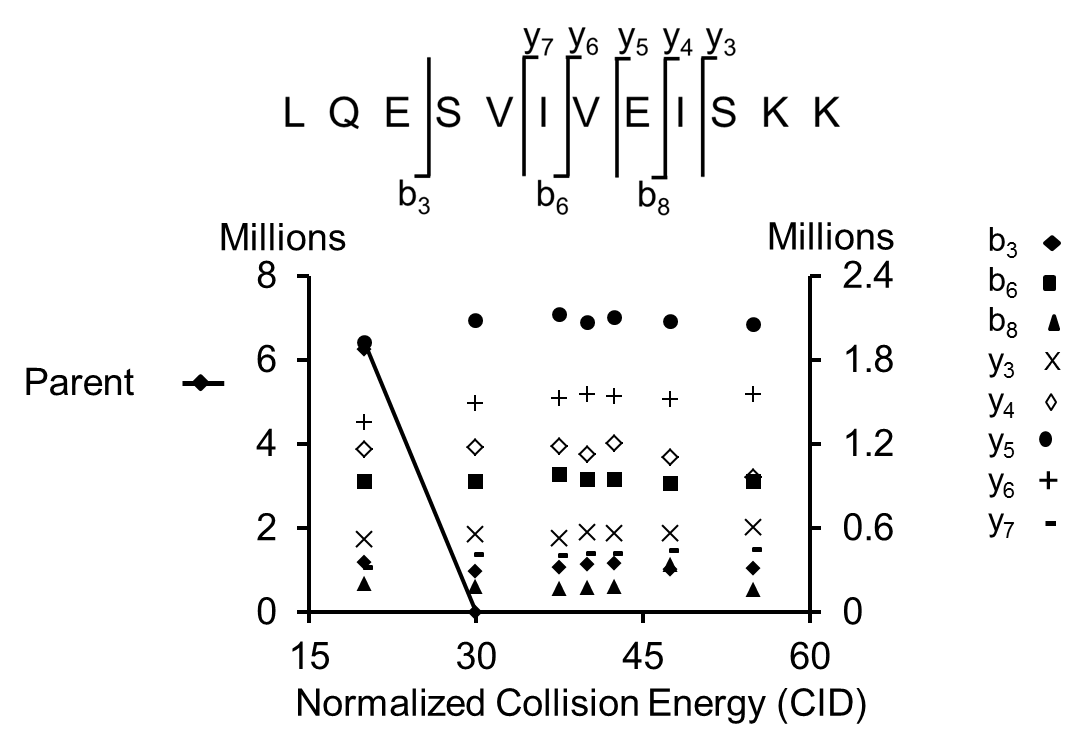

Supplement: File S1 — Inspection of Fragmentation Energy. (DOCX) [file pone.0091537.s001.docx]
