# Supplementary material for: Analysis of Isotopic Labeling in Peptide Fragments by Tandem Mass Spectrometry
Source: PLoS One. 2014 Mar 13;9(3):e91537. doi: 10.1371/journal.pone.0091537 (PMC3953442; doi:10.1371/journal.pone.0091537)
Supplement: File S2 — Peptide Sequence and Precursor Data. (DOCX) [file pone.0091537.s002.docx]

**Analysis of isotopic labeling in peptide fragments by tandem mass spectrometry**

**Doug K. Allen*, Bradley S. Evans and Igor G. L. Libourel**

**File S2: Peptide Sequence and Precursor Data**

Peptide Description:

| **Sequence** | **Charge (z)** | **Observed *m*/*z*** |
| --- | --- | --- |
| SRDPIYSNK | 2 | 540.27 |
| SQQLQNLR | 2 | 493.77 |
| LQESVIVEISKK | 3 | 458.26 |
| FQTLFK | 2 | 392.21 |

Ratio of ^13^C_0_ to ^13^C_1_; Precursor ion isotopic distribution was extracted for these four peptides, quantified and the ratio calculated:

| **Method** | **m/z** | **Prediction** | **Replicate 1** | **Replicate 2** | **Replicate 3** | **Average** | **Standard Deviation** |
| --- | --- | --- | --- | --- | --- | --- | --- |
| HCD | 458.26 | 1.36 | 1.35 | 1.39 | 1.37 | 1.37 | 0.02 |
| CID | 458.26 | 1.36 | 1.34 | 1.36 | 1.37 | 1.36 | 0.02 |
| HCD | 392.21 | 2.17 | 2.17 | 2.31 | 2.14 | 2.21 | 0.09 |
| CID | 392.21 | 2.17 | 2.19 | 2.10 | 2.23 | 2.17 | 0.06 |
| HCD | 493.77 | 1.99 | 2.18 | 2.14 | 2.07 | 2.13 | 0.06 |
| CID | 493.77 | 1.99 | 1.98 | 2.02 | 2.02 | 2.01 | 0.02 |
| HCD | 540.27 | 1.77 | 1.82 | 1.73 | 1.63 | 1.73 | 0.10 |
| CID | 540.27 | 1.77 | 1.81 | 1.78 | 1.77 | 1.79 | 0.02 |

As indicated the predicted and average values from the replicates agree to within the precision of the measurements for these peptides, therefore no significant bias in the MS^1^ peptides were observed within this set.
