# Supplementary material for: Analysis of Isotopic Labeling in Peptide Fragments by Tandem Mass Spectrometry
Source: PLoS One. 2014 Mar 13;9(3):e91537. doi: 10.1371/journal.pone.0091537 (PMC3953442; doi:10.1371/journal.pone.0091537)

**Analysis of isotopic labeling in peptide fragments by tandem mass spectrometry**

**Doug K. Allen*, Bradley S. Evans and Igor G. L. Libourel**

**File S3: Incomplete CID Fragmentation**

^13^C-labeled peptides were fragmented with CID as described in the main text. The incomplete fragmentation is indicated by the remaining parent peptide that can be observed in MS^2^. The isotopologues that are not fragmented are predominantly the higher *m*/*z* values and will result in product ions in MS^2^ that have lower ^13^C incorporation than if the entire parent had been completely fragmented. Relative abundance is plotted versus isotopologue *m*/*z*.


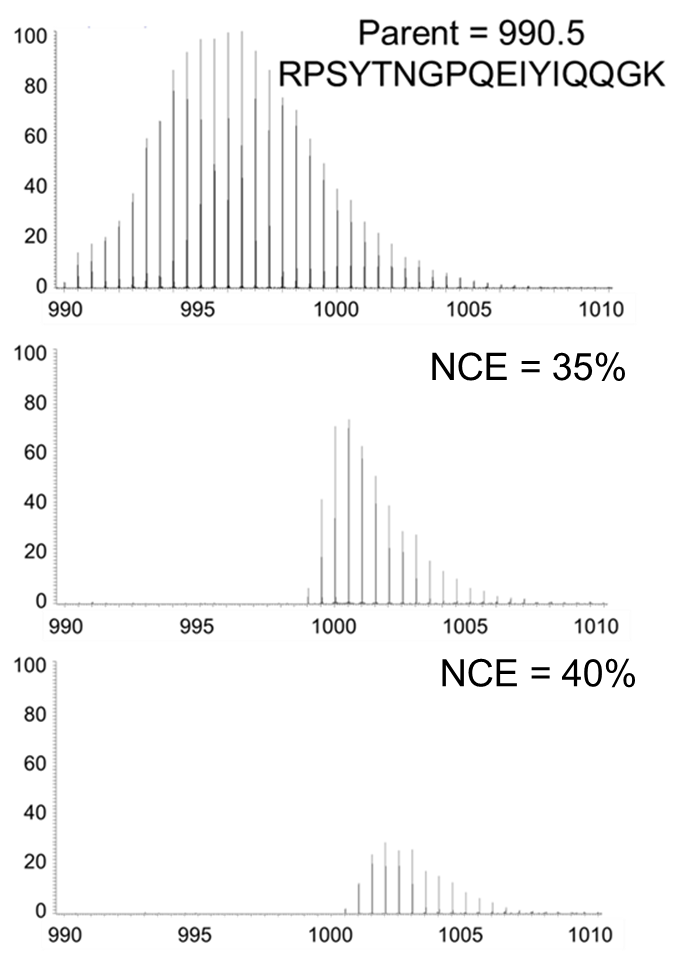

Supplement: File S3 — Incomplete CID Fragmentation. (DOCX) [file pone.0091537.s003.docx]
