# Supplementary material for: Analysis of Isotopic Labeling in Peptide Fragments by Tandem Mass Spectrometry
Source: PLoS One. 2014 Mar 13;9(3):e91537. doi: 10.1371/journal.pone.0091537 (PMC3953442; doi:10.1371/journal.pone.0091537)
Supplement: File S4 — FTMS and ITMS Comparison. (DOCX) [file pone.0091537.s004.docx]

**Analysis of isotopic labeling in peptide fragments by tandem mass spectrometry**

**Doug K. Allen*, Bradley S. Evans and Igor G. L. Libourel**

**File S4: FTMS and ITMS Comparison**


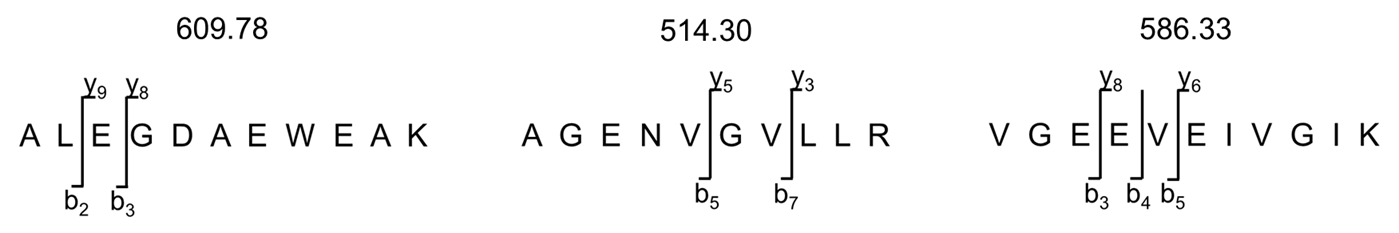


FTMS data summarized as average labeling for three peptides fragmented at multiple CID energies measured in MS^2^. Statistics represent two samples each with evaluation at three scans nearest the apex of the eluted peptide peak.

| **Parent Mass & Products** | **0** | **15** | **20** | **25** | **35** |
| --- | --- | --- | --- | --- | --- |
| **609.78** | 7.14±0.03 | 8.33±0.13 | 11.43±0.82 | --- | --- |
| **y_8_** | --- | 3.68±0.29 | 5.60±0.19 | --- | 6.77±0.09 |
| **b_3_** |  | 3.72±0.99 | 5.66±0.67 | --- | 6.12±0.25 |
|  |  |  |  |  |  |
| **514.3** | 7.07±0.03 | 8.54±0.19 | 11.88±0.14 | 14.53±0.08 | --- |
| **y_5_** | --- | 3.40±0.16 | 5.22±0.09 | 6.21±0.06 | 6.95±0.07 |
| **b_5_** | --- | 3.74±0.34 | 5.44±0.19 | 6.13±0.14 | 6.45±0.06 |
| **y_3_** | --- | 3.76±0.33 | 5.35±0.16 | 6.11±0.11 | 6.60±0.11 |
| **b_7_** | --- | 4.44±0.48 | 5.81±0.15 | 6.19±0.25 | 6.12±0.17 |
|  |  |  |  |  |  |
| **586.33** | 7.09±0.01 | 8.01±0.17 | 10.24±1.54 | 13.31±0.04 | --- |
| **y_6_** | --- | 2.89±0.20 | 5.08±0.09 | 6.07±0.12 | 6.57±0.32 |
| **b_5_** | --- | 3.03±0.28 | 5.36±0.20 | 6.00±0.37 | 5.94±0.06 |
| **y_8_** | --- | 2.78±0.19 | 5.04±0.13 | 6.10±0.10 | 6.69±0.10 |
| **b_3_** | --- | 2.16±1.12 | 4.89±0.49 | 5.57±0.58 | 6.31±0.36 |

ITMS data summarized as average labeling for three peptides fragmented at multiple CID energies measured in MS^2^. Statistics represent two samples each with evaluation at three scans nearest the apex of the eluted peptide peak.

| **Parent Mass & Products** | **0** | **15** | **20** | **25** | **35** |
| --- | --- | --- | --- | --- | --- |
| **609.78** | 7.14±0.03 | 8.33±0.13 | 11.43±0.82 | --- | --- |
| **y_8_** | --- | 4.20±0.26 | 6.97±0.24 | --- | 7.19±0.19 |
| **b_3_** | --- | 4.81±0.74 | 7.80±0.91 | --- | 6.96±1.65 |
|  |  |  |  |  |  |
| **514.3** | 7.07±0.03 | 8.54±0.19 | 11.88±0.14 | 14.53±0.08 | --- |
| **y_5_** | --- | 4.20±0.24 | 6.89±0.15 | 7.15±0.06 | 7.16±0.06 |
| **b_5_** | --- | 4.35±0.38 | 7.29±0.21 | 7.44±0.26 | 7.53±0.34 |
| **y_3_** | --- | 4.68±0.45 | 7.07±0.22 | 6.97±0.25 | 7.27±0.29 |
| **b_7_** | --- | 4.79±0.41 | 7.22±0.20 | 7.58±0.38 | 7.23±0.67 |
|  |  |  |  |  |  |
| **586.33** | 7.09±0.01 | 8.01±0.17 | 10.24±1.54 | 13.31±0.04 | --- |
| **y_6_** | --- | 4.28±0.40 | 6.73±0.30 | 7.34±0.18 | 7.13±0.29 |
| **b_5_** | --- | 3.84±0.59 | 7.50±0.49 | 7.10±0.60 | 7.16±0.95 |
| **y_8_** | --- | 3.95±0.37 | 6.38±0.27 | 7.09±0.30 | 7.35±0.22 |
| **b_3_** | --- | 2.90±1.49 | 7.22±0.99 | 7.12±0.76 | 8.03±1.04 |
