# Supplementary material for: Analysis of Isotopic Labeling in Peptide Fragments by Tandem Mass Spectrometry
Source: PLoS One. 2014 Mar 13;9(3):e91537. doi: 10.1371/journal.pone.0091537 (PMC3953442; doi:10.1371/journal.pone.0091537)

**Analysis of isotopic labeling in peptide fragments by tandem mass spectrometry**

**Doug K. Allen*, Bradley S. Evans and Igor G. L. Libourel**

**File S5: EF-Tu Peptide used to Probe Automated Gain Control**

The doubly charged ion at 690.84 was identified as A46-K57 of *E. coli* EF-Tu-1 or EF-Tu-2 by MASCOT search and manual inspection of the MS/MS spectrum. EF-Tu-1 and EF-Tu-2 are isoforms that differ at a single position so the source of the peptide is ambiguous. The annotated MS/MS spectrum is shown below.


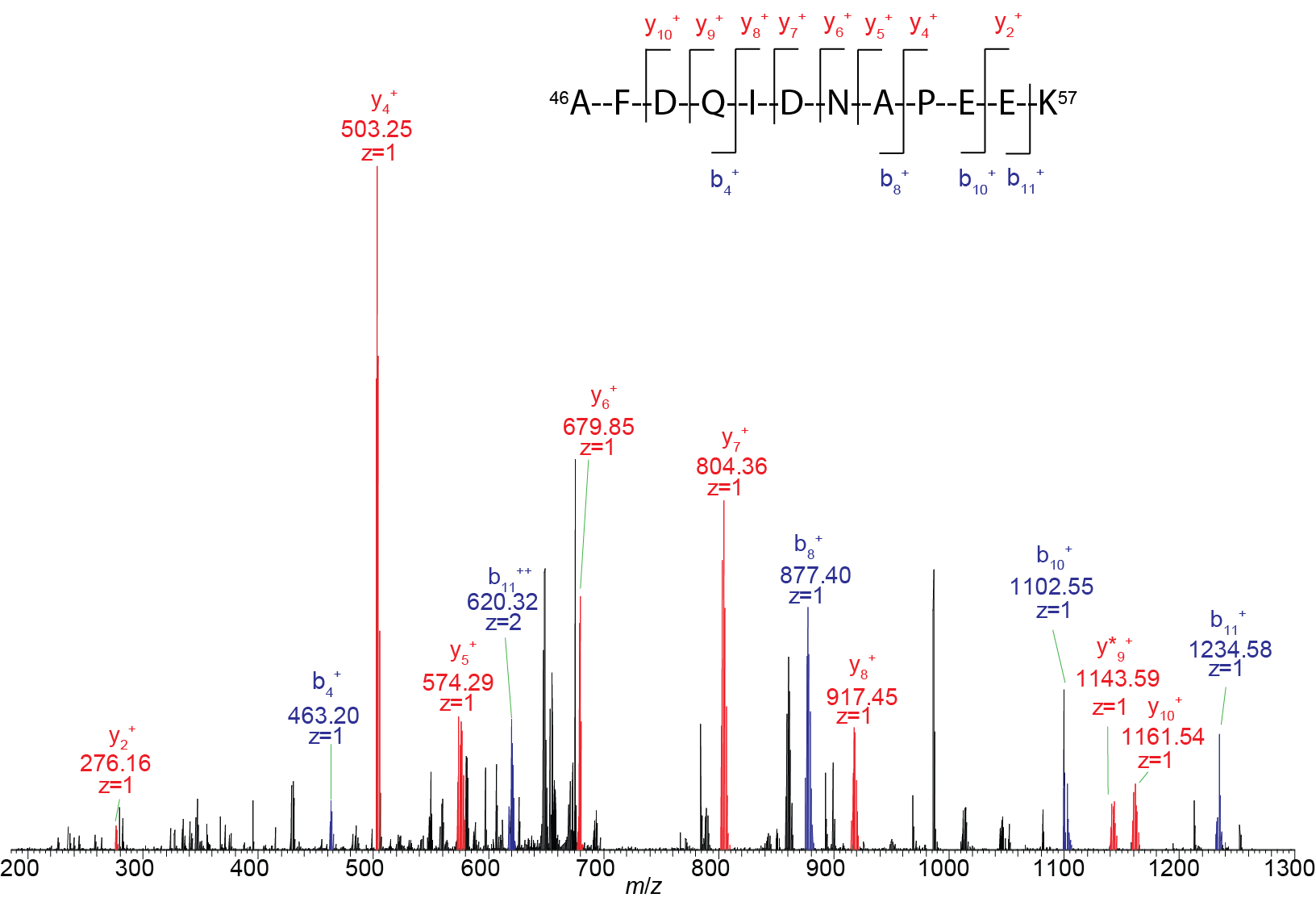

Supplement: File S5 — EF-Tu Peptide used to Probe Automated Gain Control. (DOCX) [file pone.0091537.s005.docx]
