# Supplementary material for: Analysis of Isotopic Labeling in Peptide Fragments by Tandem Mass Spectrometry
Source: PLoS One. 2014 Mar 13;9(3):e91537. doi: 10.1371/journal.pone.0091537 (PMC3953442; doi:10.1371/journal.pone.0091537)

**Analysis of isotopic labeling in peptide fragments by tandem mass spectrometry**

**Doug K. Allen*, Bradley S. Evans and Igor G. L. Libourel**

**File S6: Comparison of the Ratio of Measured Fragment Isotopologues**

Abundances of the product ion were plotted as the ratio of monoisotopic peak to monoisotopic +1 (peptide: *m*/*z* 392.21; sequence: FQTLFK). The ratio of intensities, is not based upon measurements at higher *m*/*z* values (i.e. as would be true for relative abundances) therefore the ratio will not consider the absence of *m*/*z* values that were present at background levels. This comparison would indicate the discrepancy for CID values were a consequence of low signal to noise. The ratio is highest for the low energy CID input, indicating preferential fragmentation as described in the main text. The ratios provide a pattern similar to the direct plot of the monoisotopic peak intensities in Figure 6 and do not imply that differences in abundance are an issue.


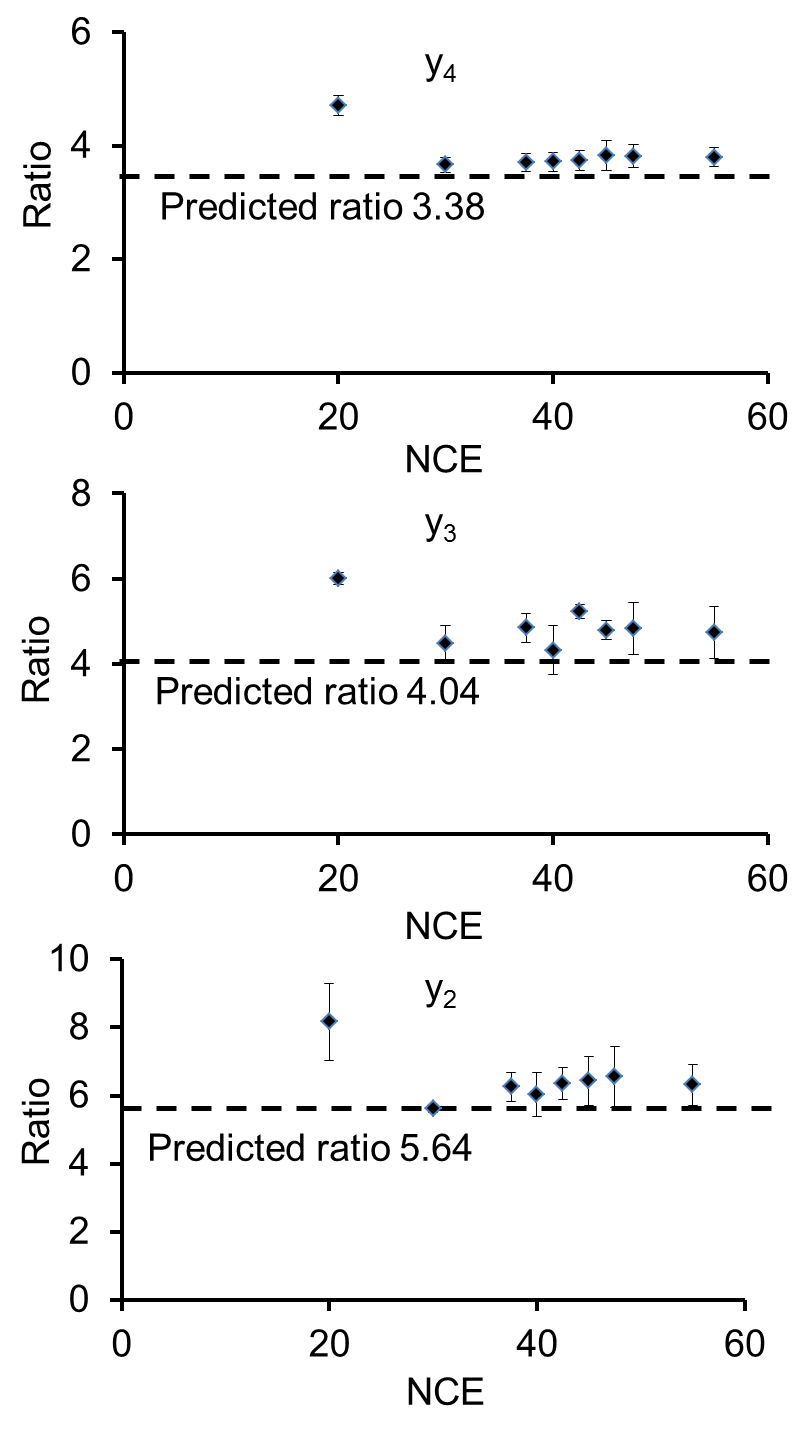

Supplement: File S6 — Comparison of the Ratio of Measured Fragment Isotopologues. (DOCX) [file pone.0091537.s006.docx]
