# Supplementary material for: Analysis of Isotopic Labeling in Peptide Fragments by Tandem Mass Spectrometry
Source: PLoS One. 2014 Mar 13;9(3):e91537. doi: 10.1371/journal.pone.0091537 (PMC3953442; doi:10.1371/journal.pone.0091537)

**Analysis of isotopic labeling in peptide fragments by tandem mass spectrometry**

**Doug K. Allen*, Bradley S. Evans and Igor G. L. Libourel**

**File S7: Fragment Abundances Contribute to Variability**

The relative abundances for three fragments from the same peptide obtained from ^13^C-labeled soybean protein were measured in multiple scan events (*m*/*z* 990.5, n=3). Greater fragment ion intensities resulted in improved precision, as indicated by reduced standard deviations for y adducts consistent with previous findings.


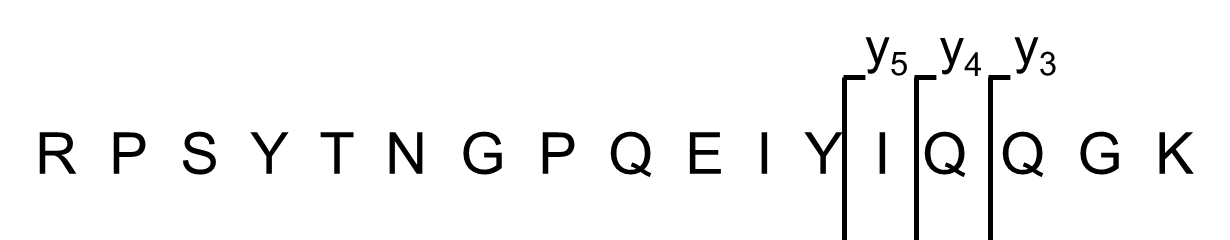


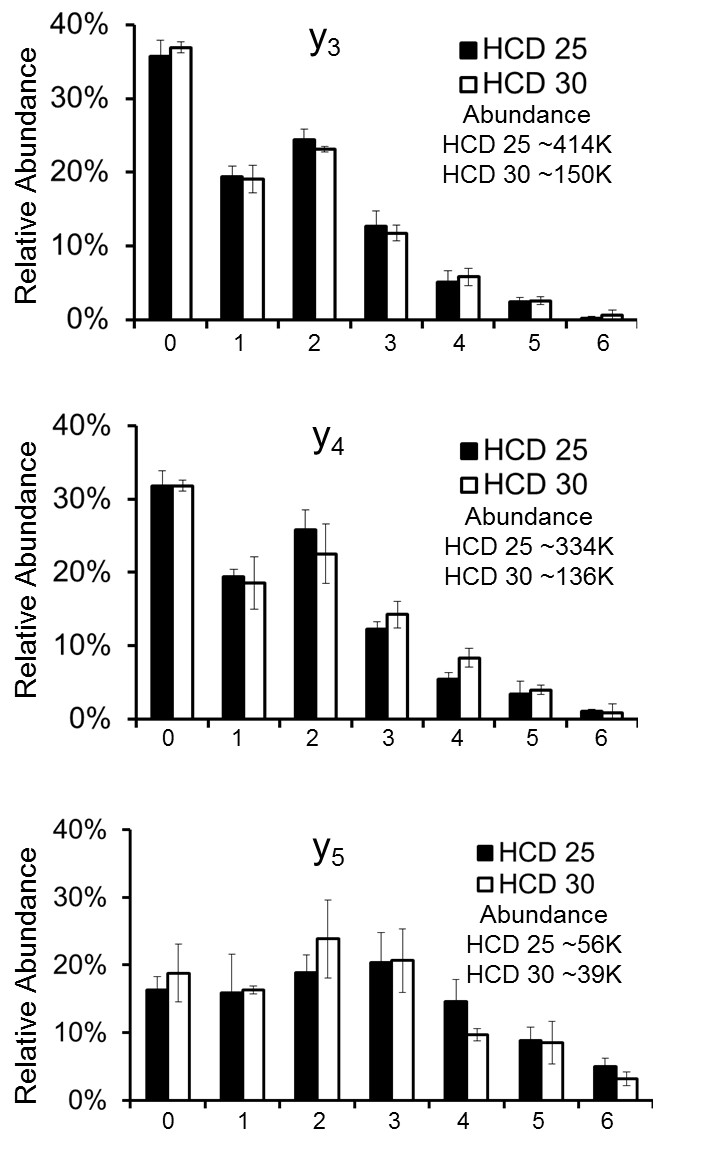

Supplement: File S7 — Fragment Abundances Contribute to Variability. (DOCX) [file pone.0091537.s007.docx]
