# Supplementary material for: Analysis of Isotopic Labeling in Peptide Fragments by Tandem Mass Spectrometry
Source: PLoS One. 2014 Mar 13;9(3):e91537. doi: 10.1371/journal.pone.0091537 (PMC3953442; doi:10.1371/journal.pone.0091537)
Supplement: File S8 — Impact of Isolation Window on Isotopic Measurements. (DOCX) [file pone.0091537.s008.docx]

**Analysis of isotopic labeling in peptide fragments by tandem mass spectrometry**

**Doug K. Allen*, Bradley S. Evans and Igor G. L. Libourel**

**File S8: Impact of Isolation Window on Isotopic Measurements**

The isolation window was varied to examine the impact on the fragmentation of the precursor isotopic distribution. Isolation windows of: 20, 25, 35, and 50 were used along with a doubly charged peptide: RFYLAGNQEQEFLK, monoisotopic *m*/*z*= 871.94. The isolation/activation was centered on *m*/*z* 880. Each isolation window was evaluated by extracting the relative intensities of the isotopologues frm three replicate scans. The entire envelope was evaluated and extends from *m*/*z* 871 to 895. The wider isolation windows did not result in symmetric fragmentation of the isotopic distribution. The relative abundances of isotopologues are indicated in the supplementary table.

|  | **Scan 1**  **Isolation Window** | | | | **Scan 2**  **Isolation Window** | | | | **Scan 3**  **Isolation Window** | | | |
| --- | --- | --- | --- | --- | --- | --- | --- | --- | --- | --- | --- | --- |
| **Mass** | 20 | 25 | 35 | 50 | 20 | 25 | 35 | 50 | 20 | 25 | 35 | 50 |
|  |  |  |  |  |  |  |  |  |  |  |  |  |
| **872.0** | 0 | 0 | 0 | 0 | 0 | 0 | 0 | 0 | 0 | 0 | 0 | 0 |
| **872.5** | 0 | 0 | 0 | 0 | 0 | 0 | 0 | 0 | 0 | 0 | 0 | 0 |
| **873.0** | 0 | 0 | 0 | 0 | 0 | 0 | 0 | 0 | 0 | 0 | 0 | 0 |
| **873.5** | 0 | 0 | 0 | 0 | 0 | 0 | 0 | 0 | 0 | 0 | 0 | 0 |
| **874.0** | 0 | 0 | 0 | 0 | 0 | 0 | 0 | 0 | 0 | 0 | 0 | 0 |
| **874.5** | 0 | 0 | 0 | 0 | 0 | 0 | 0 | 0 | 0 | 0 | 0 | 0 |
| **875.0** | 0 | 0 | 0 | 0 | 0 | 0 | 0 | 0 | 0 | 0 | 0 | 0 |
| **875.5** | 0 | 0 | 0 | 0 | 0 | 0 | 0 | 0 | 0 | 0 | 0 | 0 |
| **876.0** | 0 | 0 | 0 | 0 | 0 | 0 | 0 | 0 | 0 | 0 | 0 | 0 |
| **876.5** | 0 | 0 | 0 | 0 | 0 | 0 | 0 | 0 | 0 | 0 | 0 | 0 |
| **877.0** | 0 | 0 | 0 | 0 | 0 | 0 | 0 | 0 | 0 | 0 | 0 | 0 |
| **877.5** | 0 | 0 | 0 | 0 | 0 | 0 | 0 | 0 | 0 | 0 | 0 | 0 |
| **878.0** | 0 | 0 | 0 | 0 | 0 | 0 | 0 | 0 | 0 | 0 | 0 | 0 |
| **878.5** | 0 | 0 | 0 | 0 | 0 | 0 | 0 | 0 | 0 | 0 | 0 | 0 |
| **879.0** | 0 | 0 | 0 | 0 | 0 | 0 | 0 | 0 | 0 | 0 | 0 | 0 |
| **879.5** | 0 | 0 | 0 | 0 | 0 | 0 | 0 | 0 | 0 | 0 | 0 | 0 |
| **880.0** | 0 | 0 | 0 | 0 | 0 | 0 | 0 | 0 | 0 | 0 | 0 | 0 |
| **880.5** | 0 | 0 | 0 | 0 | 0 | 0 | 0 | 0 | 0 | 0 | 0 | 0 |
| **881.0** | 0 | 0 | 0 | 0 | 0 | 0 | 0 | 0 | 0 | 0 | 0 | 0 |
| **881.5** | 0.7% | 1.1% | 0 | 0.1% | 0 | 0 | 0 | 0.2% | 0 | 0 | 0 | 0 |
| **882.0** | 2.5% | 2.9% | 0 | 0.6% | 0 | 0 | 0 | 0.2% | 0 | 0 | 0 | 0.6% |
| **882.5** | 5.8% | 8.2% | 0 | 3.7% | 0 | 0 | 0 | 0.6% | 0 | 0 | 0 | 0.8% |
| **883.0** | 11.3% | 13.3% | 3.3% | 8.8% | 0 | 0 | 0 | 1.4% | 0 | 0 | 0 | 0.8% |
| **883.5** | 15.6% | 13.1% | 9.6% | 12.0% | 0 | 0 | 1.2% | 2.9% | 0 | 0 | 0 | 0.8% |
| **884.0** | 15.6% | 12.3% | 12.4% | 12.3% | 0.8% | 0 | 1.8% | 6.1% | 0 | 0 | 0.8% | 1.7% |
| **884.5** | 13.7% | 10.2% | 12.1% | 10.3% | 2.7% | 1.0% | 2.8% | 8.8% | 0 | 0.9% | 0.0% | 0.5% |
| **885.0** | 11.6% | 8.5% | 11.3% | 9.6% | 6.3% | 1.9% | 5.6% | 9.7% | 0 | 2.1% | 1.0% | 2.3% |
| **885.5** | 9.9% | 7.2% | 9.8% | 7.7% | 15.9% | 1.7% | 8.1% | 11.9% | 10.0% | 3.4% | 2.1% | 3.9% |
| **886.0** | 7.6% | 5.9% | 8.3% | 6.8% | 14.9% | 2.5% | 8.6% | 10.6% | 0.0% | 6.0% | 2.9% | 4.6% |
| **886.5** | 4.7% | 5.0% | 6.9% | 6.1% | 16.1% | 7.2% | 12.4% | 7.6% | 15.2% | 11.3% | 4.7% | 8.0% |
| **887.0** | 1.1% | 4.1% | 5.4% | 4.8% | 15.9% | 7.5% | 10.4% | 8.3% | 15.7% | 12.4% | 7.3% | 7.8% |
| **887.5** | 0.1% | 3.2% | 4.7% | 4.2% | 17.4% | 11.8% | 11.4% | 7.2% | 30.7% | 14.1% | 8.9% | 9.8% |
| **888.0** | 0 | 2.5% | 3.9% | 3.1% | 9.9% | 9.5% | 8.5% | 4.8% | 28.4% | 14.4% | 15.1% | 9.4% |
| **888.5** | 0 | 1.8% | 2.7% | 2.2% | 0 | 16.0% | 8.6% | 4.1% | 0 | 10.8% | 11.1% | 9.6% |
| **889.0** | 0 | 0.6% | 2.5% | 1.9% | 0 | 13.6% | 7.9% | 4.4% | 0 | 8.3% | 8.9% | 9.8% |
| **889.5** | 0 | 0 | 1.7% | 1.5% | 0 | 11.4% | 5.6% | 2.8% | 0 | 7.0% | 10.4% | 6.6% |
| **890.0** | 0 | 0 | 1.6% | 1.2% | 0 | 11.6% | 0.0% | 2.6% | 0 | 7.2% | 5.5% | 7.6% |
| **890.5** | 0 | 0 | 1.3% | 0.8% | 0 | 4.2% | 0.0% | 1.8% | 0 | 2.0% | 6.0% | 4.4% |
| **891.0** | 0 | 0 | 0.7% | 0.6% | 0 | 0 | 3.3% | 1.4% | 0 | 0 | 4.9% | 3.5% |
| **891.5** | 0 | 0 | 0.7% | 0.6% | 0 | 0 | 0.0% | 1.4% | 0 | 0 | 2.9% | 2.5% |
| **892.0** | 0 | 0 | 0.3% | 0.3% | 0 | 0 | 2.5% | 0.9% | 0 | 0 | 2.6% | 2.3% |
| **892.5** | 0 | 0 | 0.3% | 0.2% | 0 | 0 | 0.0% | 0.3% | 0 | 0 | 1.7% | 1.4% |
| **893.0** | 0 | 0 | 0.2% | 0.2% | 0 | 0 | 1.3% | 0 | 0 | 0 | 1.0% | 1.3% |
| **893.5** | 0 | 0 | 0.1% | 0.2% | 0 | 0 | 0 | 0 | 0 | 0 | 0.9% | 0 |
| **894.0** | 0 | 0 | 0.1% | 0.1% | 0 | 0 | 0 | 0 | 0 | 0 | 1.3% | 0 |
| **894.5** | 0 | 0 | 0.1% | 0.1% | 0 | 0 | 0 | 0 | 0 | 0 | 0 | 0 |
| **895.0** | 0 | 0 | 0.0% | 0.0% | 0 | 0 | 0 | 0 | 0 | 0 | 0 | 0 |

Next, the average labeling was calculated between the *m*/*z* 871 to 890 that would be isolated by all windows considered. For the wider isolation windows, the defined isotopic distribution was more centered because the window was much larger, while for the narrower isolation windows the isotopic envelope approached the periphery of the isolation window. Nonetheless, the average labeling was not statistically different as indicated in the supplementary table.

| **Isolation window** | **Average labeling per carbon** | **Standard deviation** |
| --- | --- | --- |
| **20** | 35.0% | 3.8% |
| **25** | 37.2% | 5.1% |
| **35** | 38.3% | 3.8% |
| **50** | 36.7% | 3.6% |
